# A Quantitative Clinicopathological Signature for Predicting Recurrence Risk of Pancreatic Ductal Adenocarcinoma After Radical Resection

**DOI:** 10.3389/fonc.2019.01197

**Published:** 2019-11-12

**Authors:** Chaobin He, Xin Huang, Yu Zhang, Zhiyuan Cai, Xiaojun Lin, Shengping Li

**Affiliations:** ^1^State Key Laboratory of Oncology in South China, Department of Hepatobiliary and Pancreatic Surgery, Collaborative Innovation Center for Cancer Medicine, Sun Yat-sen University Cancer Center, Guangzhou, China; ^2^State Key Laboratory of Ophthalmology, Zhongshan Ophthalmic Center, Sun Yat-sen University, Guangzhou, China

**Keywords:** pancreatic ductal adenocarcinoma, recurrence, pattern, timing, predictor

## Abstract

Recurrence and distant metastases were main reasons of unfavorable outcomes for patients with pancreatic ductal adenocarcinoma (PDAC) after surgery. The aim of this study was to describe the patterns, timing, and predictors of recurrence or metastasis in PDAC patients after curative surgery. Patients with PDAC who underwent radical pancreatectomy were included. Associations between clinicopathological and radiological characteristics and specific pattern of progression were investigated. Least absolute shrinkage and selection operator (LASSO) and Cox regression were applied to assess the prognostic factors for overall survival (OS) and progression-free survival (PFS). A total of 302 patients were included into present study, and 173 patients were documented as recurrence after a median survival of 24.7 months. More than half of patients recurred after 12 months after surgery, and the liver was the most common metastatic site. Decreased time interval to progression, elevated carbohydrate antigen 19-9 (CA19-9) level, and lymph node (LN)16 metastasis were independent predictors for reduced OS. Independent prognostic factors for PFS included elevated carcinoembryonic antigen (CEA) level, local progression, liver or lung-only metastasis, local + distant progression, multiple metastases, LN16 metastasis, imaging tumor size, chemotherapy, and tumor–node–metastasis (TNM) stage. The predictive system showed valuable prediction performance with values of concordance indexes (C-indexes) and the area under the receiver operating characteristic curve (AUC) over 0.80. Different survival curves and predictive factors for specific patterns of disease progression suggested the biological heterogeneity, providing new versions into personal management of recurrence in PDAC patients after surgery.

## Introduction

Pancreatic ductal adenocarcinoma (PDAC) is a lethal disease and is predicted to become the second leading cause of cancer-specific death by 2030 (1). Surgery followed by adjuvant chemotherapy has been widely established as the best mean to obtain longer survival. However, this combination therapy can only be applied to 20% of patients, whereas most patients suffered from locally advanced or metastatic diseases, owing to the lack of early clinical symptoms and effective screening methods. Moreover, even after curative resection, up to 80% of patients suffered from recurrence soon after surgery (2–4), and the 5-year survival rate was <6% (5).

Progression had a truly negative effect on prognoses of patients with PDAC. However, the variations of biological behaviors and clinicopathological factors of tumors would contribute to different patterns and timing of progression even when diseases were classified as the same stages. Although multiple studies illustrated the risk factors of progression, such as resection margin status and lymph node (LN) metastasis (6, 7), the relationship between the prognosis and progression was rarely evaluated for patients with PDAC. The prognosis might be changing among patients with different patterns and timing of progression, whereas significant heterogeneity existed among the current reports regarding patterns and timing of recurrence owing to the small sample sizes and limited period of follow-up (8, 9). Understanding both the risk factors and the patterns of progression of PDAC patients can provide an insight into optimization of the treatment, as well as the surveillance strategies. Although recurrence was associated with decreased survival, whether the sites and timing of recurrence had different influences on survival remained controversial. Thus, the aim of this study was to evaluate the risk factors for different patterns of recurrences and compare the survival differences in PDAC patients with varied patterns or timing of disease progression.

## Materials and Methods

### Patients

This study included consecutive patients with PDAC who underwent surgical resection at Sun Yat-sen University Cancer Center (SYSUCC) between 2008 and 2018. Excluded patients were those with metastatic diseases detected at diagnosis by radiological examination, such as computed tomography (CT) and magnetic resonance imaging (MRI). Positron emission tomography/CT (PET/CT) and diagnostic laparoscopy were also selectively performed to detect metastases on the basis of the recommendation of the pancreatic multidisciplinary team. The resection margin for radical margin was defined as 1.5–2 mm, which was the same as that of previous studies (10, 11). Excluded were also patients with microscopic or macroscopic incomplete resection, a history of secondary tumors, period of follow-up <1 year, and missing follow-up records.

### Data Collection

Resectability was judged on the basis of CT or MRI, and staging was determined by the pathological factors in accordance with the 8th edition of American Joint Committee on Cancer staging system (12, 13). A team specialized in pancreatic surgery performed all radical pancreatic resection. An experienced pancreatic pathologist assessed all the surgical specimens, made the diagnosis of PDAC, and described the pathological variables, including tumor site, tumor size, tumor differentiation, T-stage, LN status (N-stage), LN total number, positive LN number, macrovascular invasion, microvascular invasion, lymph vessel invasion, perineural invasion, adjacent organ invasion, and satellite foci. LN ratio (LNR) was defined as the number of LNs with metastases divided by the total number of excised LNs. Several radiological variables, including imaging tumor size, LN metastasis, vascular invasion, and LN size, were analyzed. Inflammation-based indexes, such as neutrophil-to-lymphocyte ratio (NLR), platelet-to-lymphocyte ratio (PLR), modified Glasgow Prognostic Score (mGPS), prognostic nutritional index (PNI), prognostic index (PI), and systemic immune-inflammation index (SII), were included in this study and calculated according to previous studies (14, 15). Clinical data were also analyzed in this study, including age, gender, white blood cell (WBC) count, C-reactive protein (CRP), albumin (ALB), serum levels of carbohydrate antigen 19-9 (CA19-9), and carcinoembryonic antigen (CEA).

### Follow-Up and Recurrence

The follow-up of patients occurred at the outpatient clinic of our hospital. In general, follow-up strategies consisted of regular chest CT, abdominal CT, and CA19-9 test at least every 2 months during the first year after surgical resection and every 3 months thereafter. Occasional additional imaging modalities, such as MRI and PET/CT, were selectively performed to determine patterns of recurrence. Follow-up data were retrieved at the end of May 2019. The categories of regression patterns in the study conducted by Groot et al. (4) were adopted in this study. When considering patterns of recurrence, only the first location of recurrence was documented, and local recurrence and distant recurrence were registered, separately. In addition, distant recurrences were judged as “liver-only” and “lung-only” recurrences for the isolated hepatic and pulmonic recurrence, respectively, and “others” for isolated recurrence in other less common locations. If both local recurrence and isolated distant metastasis occurred or multiple distant metastases were detected at the same time, recurrences were defined as “local + distant” and “multiple” recurrences, respectively.

### Survival Outcomes and Statistical Analysis

Progression-free survival (PFS) and overall survival (OS) were defined as the duration from the date of surgery until the date when tumor progression was diagnosed and death, respectively, or last follow-up. Post-progression survival (PPS) was defined as the time from the first recurrence to either death or last follow-up. Survival time was estimated using the Kaplan–Meier method, and the subgroup differences were compared with log-rank test. Univariate analyses were performed to describe the association between clinical, pathological, and radiological factors and specific patterns of recurrence. For PFS and OS prediction, multivariate logistic regression was conducted on the basis of clinical characteristics and pathological or radiological variables selected by least absolute shrinkage and selection operator (LASSO) logistic regression model. The prediction algorithms were further validated using receiver operating characteristic (ROC) curves. Area under the ROC curve (AUC) and concordance index (C-index) of the multi-marker algorithms were calculated and compared. Two-tailed *P* < 0.05 were considered statistically significant. All statistical analyses were conducted using R software version 3.2.5 (R Development Core Team; http://www.r-project.org).

## Results

### Patient Characteristic

From 2008 to 2018, a total of 355 patients underwent radical pancreaticoduodenectomy (PD) or distal pancreatectomy for histologically confirmed PDAC. Excluded from this cohort were 10 patients with microscopic or macroscopic incomplete resection, 12 patients with second primary tumors, and 31 patients with incomplete follow-up information. Consequently, 302 patients were included into this study. All patients were followed up at least 1 year. At the end of follow-up, 195 patients (64.6%) were alive after a median follow-up of 24.7 months (95% confidence interval [CI] 20.3–29.1) from surgery. Recurrence was documented in 173 patients (57.3%), whereas 129 patients (42.7%) had no signs of recurrence. The median follow-up time for patients with and without tumor progression was 13.8 and 40.6 months, respectively.

### Timing of Recurrence

**Figure 1:**
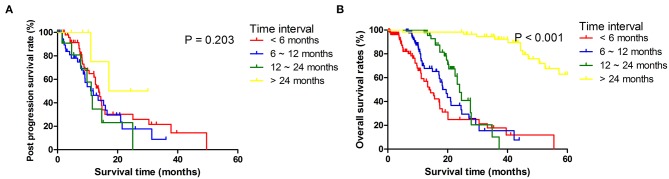
Post progression survival **(A)** and overall survival **(B)** stratified by time period to tumor progression diagnosis counted from the date of surgery.

Among 173 patients who had recurrence, 18 patients had done so within 6 months, 26 within 6–12 months, 57 within 12–24 months, and 72 beyond 24 months after surgery. There were no significant differences in ages and sexes among patients in different recurrent time groups. Primary tumors in early recurrence groups were larger, more likely to be poorly differentiated, and diagnosed at more advanced local stages. Patients with early recurrence had more often T4 tumors, more metastatic LNs, and more often para-aortic LNs (LN16) metastasis than had those in late recurrence groups (Table 1). Median PFS was 11.8 months (95% CI 10.2–15.3) for the whole cohort and 7.0 months (95% CI 6.2–8.4) for those who developed recurrences. For patients who developed recurrences, the comparisons of PPS and OS stratified by different time intervals of recurrences are shown in Figure 1. It was shown that median OS and PPS for patients who developed recurrences beyond 24 months over surgery (OS, 45.1 months, 95% CI 40.2–52.6; PPS, 17.1 months, 95% CI 11.1–17.5) were significantly longer than for those who had recurrence within 24 months since surgery. Also, patients had similar OS and PPS when their recurrences developed within 6, 6 to 12, or 12 to 24 months since surgery.

**Table 1:** Clinicopathological characteristics of patients with PDAC stratified by time of metastases.

**Characteristics**		**Diagnosis of progression**							**Characteristics**		**Diagnosis of progression**						
		***N***	**Absence**	**2–6 M**	**6–12 M**	**12–24 M**	**>24 M**	***P***			***N***	**Absence**	**2–6 M**	**6–12 M**	**12–24 M**	**>24 M**	***P***
Whole cohort		302	129	18	26	57	72		Whole cohort		302	129	18	26	57	72	
Age	≤60 years	164	74	8	12	29	41	0.670	Perineural invasion	Absence	146	70	8	13	21	34	0.287
	>60 years	138	55	10	14	28	31			Presence	156	59	10	13	36	38	
Gender	Female	119	53	7	13	25	21	0.286	Adjacent organ invasion	Absence	270	119	15	24	52	60	0.284
	Male	183	76	11	13	32	51			Presence	32	10	3	2	5	12	
Recurrence	Absence	174	129	10	11	11	13	<0.001	LNR	0	173	83	12	17	26	35	0.038
	Presence	128	0	8	15	46	59			0–0.16	66	26	1	7	17	15	
Recurrence patterns	Absence	174	129	10	11	11	13	<0.001		>0.16	63	20	5	2	14	22	
	Local	39	0	4	6	18	11		Satellite foci	Absence	287	123	18	26	55	65	0.197
	Liver only	49	0	1	5	14	29			Presence	15	6	0	0	2	7	
	Lung only	12	0	2	0	4	6		T stage	T1	82	46	5	9	8	14	0.023
	Other sites	5	0	1	3	1	0			T2	136	57	8	10	34	27	
	Local + distant	14	0	0	1	5	8			T3	57	18	3	4	12	20	
	Multiple	9	0	0	0	4	5			T4	27	8	2	3	3	11	
LN metastasis	Absence	174	83	13	17	26	35	0.035	Tumor site	Head	247	111	13	23	46	54	0.221
	Presence	128	46	5	9	31	37			Body and tail	55	18	5	3	11	18	
LN5 metastasis	Absence	300	127	18	26	57	72	0.609	TNM stage	IA	54	33	4	7	3	7	0.003
	Presence	2	2	0	0	0	0			IB	74	36	6	7	13	12	
LN6 metastasis	Absence	298	126	18	26	57	71	0.672		IIA	35	11	2	2	10	10	
	Presence	4	3	0	0	0	1			IIB	79	32	3	6	21	17	
LN7 metastasis	Absence	296	128	17	25	56	70	0.582	Imaging tumor size (cm)	III	60	17	3	4	10	26	
	Presence	6	1	1	1	1	2			≤2	104	63	6	5	13	17	0.001
LN8 metastasis	Absence	294	126	17	25	57	69	0.561		2–4	141	45	9	19	31	37	
	Presence	8	13	1	1	0	3			>4	57	21	3	2	13	18	
LN9 metastasis	Absence	292	125	17	25	57	68	0.492	Imaging LN metastasis	Absence	175	73	12	15	34	41	0.944
	Presence	10	4	1	1	0	4			Presence	127	56	6	11	23	31	
LN10 metastasis	Absence	295	127	17	26	56	69	0.566	Imaging vascular invasion	Absence	234	106	16	22	42	48	0.060
	Presence	7	2	1	0	1	3			Presence	68	23	2	4	15	24	
LN11 metastasis	Absence	294	126	18	26	56	68	0.436	Imaging LN size (cm)	≤0.5	177	72	13	16	35	41	0.884
	Presence	8	3	0	0	1	4			0.5–1	64	30	1	5	11	17	
LN12 metastasis	Absence	268	116	18	23	49	62	0.493	PI	>1	61	27	4	5	11	14	
	Presence	34	13	0	3	8	10			0	199	93	12	16	36	42	0.168
LN13 metastasis	Absence	231	103	15	21	40	52	0.473		1	84	31	6	7	19	21	
	Presence	71	26	3	5	17	20			2	19	5	0	3	2	9	
LN14 metastasis	Absence	281	122	16	26	52	65	0.402	NLR	≤3.32	197	89	13	16	36	43	0.659
	Presence	21	7	2	0	5	7			>3.32	105	40	5	10	21	29	
LN15 metastasis	Absence	294	127	18	26	56	67	0.129	dNLR	≤3.32	100	39	10	9	20	22	0.296
	Presence	8	2	0	0	1	5			>3.32	202	90	8	17	37	50	
LN16 metastasis	Absence	284	127	18	26	52	61	0.001	PLR	≤98.13	36	17	5	1	7	6	0.135
	Presence	18	2	0	0	5	11			>98.13	266	112	13	25	50	66	
LN17 metastasis	Absence	293	124	18	26	54	71	0.498	PNI	0	65	31	6	2	11	15	0.277
	Presence	9	5	0	0	3	1			1	237	98	21	24	46	57	
LN18 metastasis	Absence	296	126	18	26	54	72	0.234	SII	≤1,000	206	90	14	16	26	50	0.706
	Presence	6	3	0	0	3	0			>1,000	96	39	4	10	21	22	
Positive LN number	0	173	83	12	17	26	35	0.046	mGPS	0	202	93	12	16	38	43	0.677
	1–3	95	36	5	8	24	22			1	67	23	4	7	11	22	
	>3	34	10	1	1	7	15			2	33	13	2	3	8	7	
Pancreatic membrane invasion	Absence	184	81	15	13	36	39	0.147	WBC count	≤10	280	124	18	23	53	62	0.061
	Presence	118	48	3	13	21	33			>10	22	5	0	3	4	10	
Tumor size (cm)	≤2	88	48	6	10	9	15	0.012	ALB (g/L)	≤35	46	19	2	4	12	9	0.704
	2–4	146	60	8	10	36	32			>35	256	110	16	22	45	63	
	>4	68	21	4	6	12	25		CRP (ng/L)	≤3	202	93	12	16	38	43	0.465
Tumor differentiation	Well	2	0	0	0	1	1	0.035		>3	100	36	6	10	19	29	
	Moderate	153	72	14	12	30	25		CA19-9 (U/ml)	≤35	59	34	4	5	5	11	0.063
	Poor	147	57	4	14	26	46			>35	243	95	14	21	52	61	
Macrovascular invasion	Absence	273	120	16	23	54	60	0.161	CEA (ng/ml)	≤5	205	97	14	17	37	40	0.054
	Presence	29	9	2	3	3	12			>5	97	32	4	9	20	32	
Microvascular invasion	Absence	206	87	15	19	40	45	0.493	HBV infection	Absence	283	120	16	25	54	68	0.871
	Presence	96	42	3	7	17	27			Presence	19	9	2	2	3	4	
Lymph vessel invasion	Absence	140	65	8	12	21	34	0.296	Chemotherapy	No	160	78	10	14	21	37	0.061
	Presence	162	62	11	13	38	38			Yes	142	51	8	12	36	35	

*M, month; LN, lymph node metastasis; LNR, lymph node ratio; TNM, tumor–node–metastasis; PI, prognostic index; NLR, neutrophil-to-lymphocyte ratio; PLR, platelet-to-lymphocyte ratio; PNI, prognostic nutritional index; SII, systemic immune-inflammation index; mGPS, modified Glasgow Prognostic Score; WBC, white blood cell; ALB, albumin; CRP, C-reactive protein; CA19-9, carbohydrate antigen 19-9; CEA, carcinoembryonic antigen; HBV, hepatitis B virus; PDAC, pancreatic ductal adenocarcinoma*.

### Patterns of Recurrence

**Figure 2:**
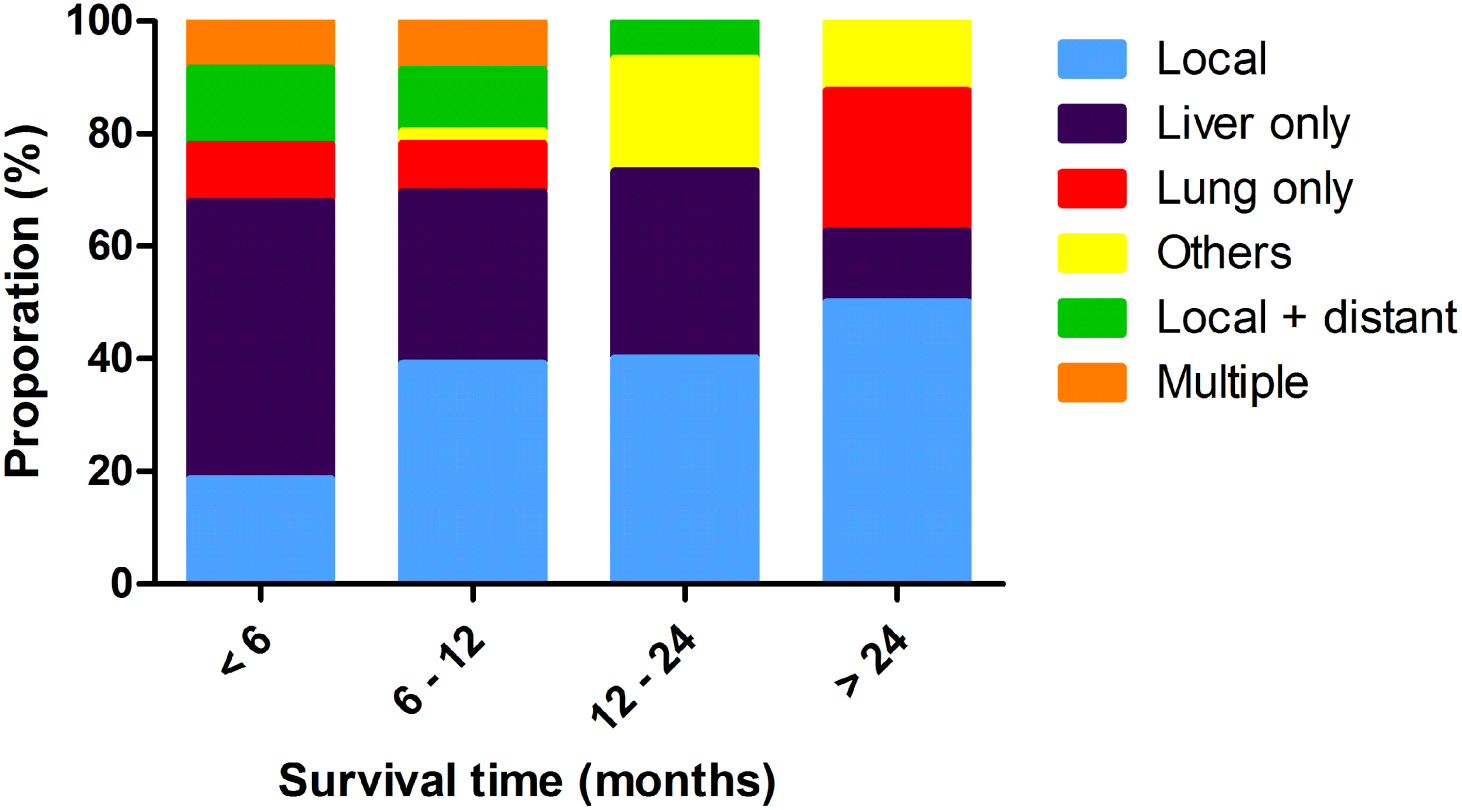
Distribution of tumor progression pattern at different time points.

Overall, there were six different patterns of recurrence for all radiological or pathological evidence of progression. Most of patients first recurred at the liver (*n* = 69, 39.9%), followed by local progression (*n* = 55, 31.8%), and lung metastases (*n* = 17, 9.8%). There were 20 (11.6%) patients who had both local and distant progression, and multiple recurrences were observed in 12 (6.9%) patients as the first progression. Liver and lung metastases were the most common distant metastases, compared with the local recurrence, and also contributed to most of the multiple progressions. The proportions of recurrence locations differed significantly at progressive time points. Distribution of these recurrent patterns is shown in Figure 2. Liver-only progressions occupied the majority of all progressions within 6 months, whereas they were responsible for just 12.5% of all recurrences after 24 months since surgery (*P* < 0.001). Also, liver-only progression diminished over time, and recurrences of other sites became more and more common 1 year later since surgery.

**Figure 3:**
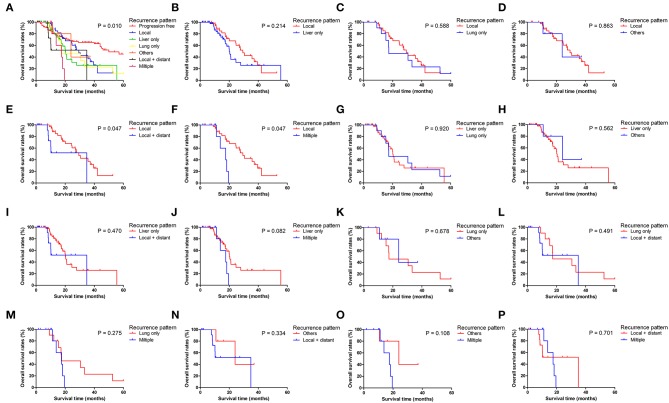
Pairwise comparison of overall survival for different tumor progression patterns. **(A)** Stratification of patients using different progression patterns of progression free, local, liver only, lung only, others, local and distant and multiple progressions. **(B–P)** Stratification of patients by comparing the following patterns of progression: local vs. liver only, local vs. lung only, local vs. others, local vs. local + distant, local vs. multiple, liver only vs. lung only, liver only vs. others, liver only vs. local + distant, liver only vs. multiple, lung only vs. others, lung only vs. local + distant, lung only vs. multiple, others vs. local + distant, others vs. multiple and local + distant vs. multiple.

**Figure 4:**
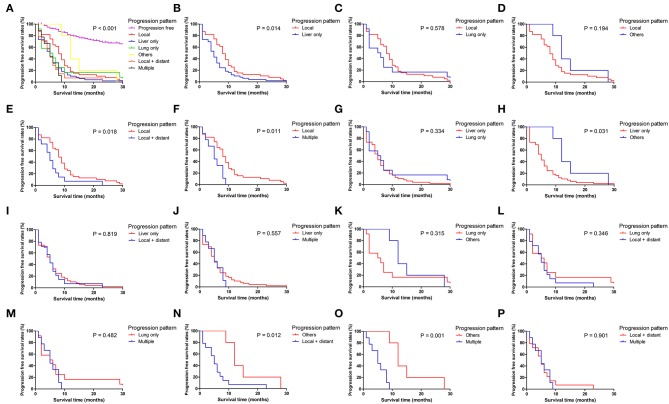
Pairwise comparison of progression-free survival for different tumor progression patterns. **(A)** Stratification of patients using different progression patterns of progression free, local, liver only, lung only, others, local and distant and multiple progressions. **(B–P)** Stratification of patients by comparing the following patterns of progression: local vs. liver only, local vs. lung only, local vs. others, local vs. local + distant, local vs. multiple, liver only vs. lung only, liver only vs. others, liver only vs. local + distant, liver only vs. multiple, lung only vs. others, lung only vs. local + distant, lung only vs. multiple, others vs. local + distant, others vs. multiple and local + distant vs. multiple.

Patients with different progression patterns had significantly different cumulative recurrence rates in different time periods after surgery (Supplement Figure 1). It was shown that cumulative rates of liver metastasis were significantly higher than those of local and other sites of progression, whereas the cumulative rates of liver, lung, and local plus distant and multiple metastases were comparable. The pairwise comparisons of OS (Figure 3), PPS (Supplement Figure 2), and PFS (Figure 4) for patients with different recurrence patterns were conducted. Median OS for patients with local recurrence (29.4 months, 95% CI 24.5–39.6) was significantly longer than that of patients with multiple progressions (17.5 months, 95% CI 11.2–19.5), whereas patients with other recurrence patterns of progression had similar OS rates. Similar results of survival comparisons were observed for PPS. In terms of PFS, patients with local (9.0 months, 95% CI 6.4–10.6) and other sites of progressions (12.7 months, 95% CI 9.1–28.7) had similar median survival, whereas they were both higher than those with other patterns of progressions.

### Risk Factors for Different Patterns of Recurrence

Results of univariate and multivariate logistic regression models for local recurrence and liver-only metastasis are shown in Tables 2, 3, respectively. Also, risk factors of lung only, other sites of metastasis, local + distant, and multiple metastases are shown in Supplement Tables 1–4, respectively. Age older than 60 years was a strong predictor for both liver-only metastasis (hazard ratio [HR] = 1.35, 95% CI 1.21–1.73, *P* = 0.031) and multiple metastases (HR = 9.82, 95% CI 1.20–80.66, *P* = 0.033). Specific stations of LN metastases were significantly associated with different patterns of progressions, including LN15 metastasis as a predictor for liver-only (HR = 6.39, 95% CI 1.29–31.52, *P* = 0.023) and local + distant metastases (HR = 8.51, 95% CI 1.27–59.11, *P* = 0.030), LN18 metastasis as a predictor for local progression (HR = 8.97, 95% CI 1.48–54.23, *P* = 0.017), LN10 metastasis as a predictor for lung-only metastasis (HR = 15.96, 95% CI 1.89–134.86, *P* = 0.011), and LN14 metastasis as a predictor for multiple metastases (HR = 7.38, 95% CI 1.61–33.74, *P* = 0.010). Patients receiving adjuvant chemotherapy had a decreased likelihood of local progression (HR = 0.18, 95% CI 0.08–0.42, *P* < 0.001) and lung-only metastasis (HR = 0.14, 95% CI 0.02–0.83, *P* = 0.031) than are those who did not receive adjuvant chemotherapy. Also, PLR was the only independent predictor for other sites of metastases (HR = 0.13, 95% CI 0.02–0.87, *P* = 0.036), and enlarged imaging LN size was found to increase the likelihood of local + distant metastases (HR = 4.57, 95% CI 1.34–15.60, *P* = 0.015).

**Table 2:** Risk factors for local recurrence in PDAC patients after surgery.

**Characteristics**		**Univariate analysis**			**Multivariate analysis**			**Characteristics**		**Univariate analysis**			**Multivariate analysis**		
		**HR**	**95%**	***P***	**HR**	**95%**	***P***			**HR**	**95%**	***P***	**HR**	**95%**	***P***
Age	≤60 years	Reference		0.951			NI	Perineural invasion	Absence	Reference		0.188			NI
	>60 years	1.02	0.52–2.01						Presence	1.59	0.80–3.16				
Gender	Female	Reference		0.897				Adjacent organ invasion	Absence	Reference		0.941			NI
	Male	1.05	0.52–2.09						Presence	0.96	0.32–2.90				
LN metastasis	Absence	Reference		0.060			NI	LNR	0	Reference					NI
	Presence	1.92	0.97–3.28						0–0.16	1.36	0.91–2.04	0.135			
LN5 metastasis	Absence	Reference					NI		>0.16	1.50	0.70–3.22	0.302			
	Presence	–						Satellite foci	Absence	Reference		0.470			NI
LN6 metastasis	Absence	Reference					NI		Presence	0.47	0.06–3.66				
	Presence	–						Tumor site	Head	Reference		0.964			NI
LN7 metastasis	Absence	Reference		0.783			NI		Body and tail	0.98	0.41–2.35				
	Presence	1.36	0.15–11.94					Imaging tumor size (cm)	≤2	Reference					NI
LN8 metastasis	Absence	Reference					NI		2–4	1.74	0.79–3.85	0.173			
	Presence	–							>4	1.32	0.48–3.67	0.600			
LN9 metastasis	Absence	Reference					NI	Imaging LN metastasis	Absence	Reference		0.213			NI
	Presence	–							Presence	1.54	0.78–3.01				
LN10 metastasis	Absence	Reference		0.913			NI	Imaging vascular invasion	Absence	Reference		0.203			NI
	Presence	1.13	0.13–9.62						Presence	1.62	0.77–3.40				
LN11 metastasis	Absence	Reference		0.315			NI	Imaging LN size (cm)	≤0.5	Reference					NI
	Presence	2.32	0.45–11.90						0.5–1	0.63	0.23–1.75	0.374			
LN12 metastasis	Absence	Reference		0.163			NI		>1	2.01	0.94–4.32	0.073			
	Presence	1.91	0.77–4.75					PI	0	Reference					NI
LN13 metastasis	Absence	Reference		0.460			NI		1	0.94	0.43–2.06	0.878			
	Presence	1.33	0.63–2.83						2	1.86	0.57–6.04	0.304			
LN14 metastasis	Absence	Reference		0.389			NI	NLR	≤3.32	Reference		0.203			NI
	Presence	1.65	0.53–5.29						>3.32	0.61	0.29–1.31				
LN15 metastasis	Absence	Reference					NI	dNLR	≤3.32	Reference		0.067			NI
	Presence	–							>3.32	0.53	0.27–1.05				
LN16 metastasis	Absence	Reference		0.814			NI	PLR	≤98.13	Reference		0.731			NI
	Presence	0.83	0.18–3.78						>98.13	1.21	0.40–3.64				
LN17 metastasis	Absence	Reference		0.870			NI	PNI	0	Reference		0.076			NI
	Presence	0.84	0.10–6.90						1	2.64	0.90–7.73				
LN18 metastasis	Absence	Reference		0.018	Reference		0.017	SII	≤1,000	Reference		0.110			NI
	Presence	7.22	1.40–37.15		8.97	1.48–54.23			>1,000	0.51	0.23–1.16				
Positive LN number	0	Reference					NI	mGPS	0	Reference					NI
	1–3	1.72	0.82–3.63	0.154					1	0.92	0.39–2.14	0.843			
	>3	1.82	0.66–5.06	0.250					2	1.21	0.43–3.41	0.720			
Pancreatic membrane invasion	Absence	Reference		0.432			NI	WBC count	≤10	Reference		0.162			NI
	Presence	0.75	0.37–1.53						>10	2.13	0.74–6.14				
Tumor size (cm)	≤2	Reference					NI	ALB (g/L)	≤35	Reference		0.977			NI
	2–4	1.17	0.52–2.64	0.711					>35	0.99	0.39–2.51				
	>4	1.35	0.53–3.44	0.537				CRP (ng/L)	≤3	Reference		0.975			NI
Tumor differentiation	Well	Reference					NI		>3	1.01	0.50–2.07				
	Moderate	1.21	0.54–2.53	0.542				CA19-9 (U/ml)	≤35	Reference		0.485			NI
	Poor	1.46	0.76–3.68	0.286					>35	1.39	0.55–3.49				
Macrovascular invasion	Absence	Reference		0.882			NI	CEA (ng/ml)	≤5	Reference		0.204			NI
	Presence	1.09	0.36–3.31						>5	1.56	0.78–3.12				
Microvascular invasion	Absence	Reference		0.555			NI	HBV infection	Absence	Reference		0.288			NI
	Presence	1.24	0.61–2.50						Presence	1.87	0.59–5.97				
Lymph vessel invasion	Absence	Reference		0.462			NI	Chemotherapy	No	Reference		0.001	Reference		<0.001
	Presence	1.21	0.59–2.74						Yes	0.19	0.08–0.43		0.18	0.08–0.42	

*PDAC, pancreatic ductal adenocarcinoma; HR, hazard ratio; LNR, lymph node ratio; LN, lymph node metastasis; NLR, neutrophil-to-lymphocyte ratio; dNLR, derived neutrophil-to-lymphocyte ratio; PLR, platelet-to-lymphocyte ratio; PNI, prognostic nutritional index; SII, systemic immune-inflammation index; mGPS, modified Glasgow Prognostic Score; WBC, white blood cell; ALB, albumin; CRP, C-reactive protein; CA19-9, carbohydrate antigen 19-9; CEA, carcinoembryonic antigen; HBV, hepatitis B virus*.

**Table 3:** Risk factors for liver metastases in PDAC patients after surgery.

**Characteristics**		**Univariate analysis**			**Multivariate analysis**			**Characteristics**		**Univariate analysis**			**Multivariate analysis**		
		**HR**	**95%**	***P***	**HR**	**95%**	***P***			**HR**	**95%**	***P***	**HR**	**95%**	***P***
Age	≤60 years	Reference		0.030	Reference		0.031	Perineural invasion	Absence	Reference		0.145			NI
	>60 years	1.32	1.11–2.23		1.35	1.21–1.73			Presence	1.59	0.85–2.97				
Gender	Female	Reference		0.922			NI	Adjacent organ invasion	Absence	Reference		0.160			NI
	Male	1.03	0.55–1.93						Presence	1.86	0.78–4.43				
LN metastasis	Absence	Reference		0.901			NI	LNR	0	Reference					NI
	Presence	1.04	0.56–1.93						0–0.16	1.311	0.90–1.92	0.165			
LN5 metastasis	Absence	Reference					NI		>0.16	1.37	0.66–2.84	0.402			
	Presence	–						Satellite foci	Absence	Reference		0.076			NI
LN6 metastasis	Absence	Reference		0.636			NI		Presence	2.76	0.90–8.47				
	Presence	1.74	0.18–17.04					Tumor site	Head	Reference		0.614			NI
LN7 metastasis	Absence	Reference		0.269			NI		Body and tail	1.22	0.57–2.62				
	Presence	2.65	0.47–14.88					Imaging tumor size (cm)	≤2	Reference					NI
LN8 metastasis	Absence	Reference		0.500			NI		2–4	1.28	0.64–2.57	0.489			
	Presence	1.75	0.34–8.95						>4	1.11	0.45–2.73	0.816			
LN9 metastasis	Absence	Reference		0.743			NI	Imaging LN metastasis	Absence	Reference		0.901			NI
	Presence	1.30	0.27–6.33						Presence	1.04	0.56–1.93				
LN10 metastasis	Absence	Reference					NI	Imaging vascular invasion	Absence	Reference		0.031			0.053
	Presence	–							Presence	2.07	1.07–4.03		2.08	0.99–4.38	
LN11 metastasis	Absence	Reference		0.773			NI	Imaging LN size (cm)	≤0.5	Reference					NI
	Presence	0.73	0.09–6.09						0.5–1	0.67	0.29–1.55	0.353			
LN12 metastasis	Absence	Reference		0.466			NI		>1	0.92	0.42–2.02	0.842			
	Presence	1.40	0.57–3.41					PI	0	Reference					NI
LN13 metastasis	Absence	Reference		0.363			NI		1	1.27	0.64–2.52	0.487			
	Presence	1.38	0.69–2.73						2	2.09	0.70–6.25	0.186			
LN14 metastasis	Absence	Reference		0.803			NI	NLR	≤3.32	Reference		0.106			NI
	Presence	0.85	0.24–3.01						>3.32	1.67	0.90–3.11				
LN15 metastasis	Absence	Reference		0.018	Reference		0.023	dNLR	≤3.32	Reference		0.087			NI
	Presence	5.53	1.34–22.93		6.39	1.29–31.52			>3.32	1.88	0.91–3.85				
LN16 metastasis	Absence	Reference		0.050	Reference		0.252	PLR	≤98.13	Reference		0.379			NI
	Presence	2.80	1.00–7.87		1.99	0.61–6.49			>98.13	1.63	0.55–4.83				
LN17 metastasis	Absence	Reference		0.623			NI	PNI	0	Reference		0.836			NI
	Presence	1.50	0.30–7.42						1	1.08	0.51–2.31				
LN18 metastasis	Absence	Reference					NI	SII	≤1,000	Reference		0.140			NI
	Presence	–							>1,000	1.61	0.86–3.02				
Positive LN number	0	Reference					NI	mGPS	0	Reference					NI
	1–3	1.53	0.75–3.11	0.245					1	1.38	0.67–2.83	0.380			
	>3	2.15	0.85–5.44	0.105					2	1.27	0.49–3.35	0.623			
Pancreatic membrane invasion	Absence	Reference		0.063			NI	WBC count	≤10	Reference		0.152			NI
	Presence	1.79	0.97–3.32						>10	2.07	0.77–5.58				
Tumor size (cm)	≤2	Reference			Reference			ALB (g/L)	≤35	Reference		0.840			NI
	2–4	2.37	1.03–5.47	0.043	1.97	0.83–4.68	0.124		>35	1.09	0.46–2.61				
	>4	2.36	0.92–6.08	0.074	1.48	0.53–4.19	0.457	CRP (ng/L)	≤3	Reference		0.359			NI
Tumor differentiation	Well	Reference					NI		>3	1.35	0.72–2.53				
	Moderate	0.11	0.01–1.83	0.123				CA19-9 (U/ml)	≤35	Reference		0.079			NI
	Poor	0.29	0.02–4.75	0.385					>35	2.39	0.90–6.32				
Macrovascular invasion	Absence	Reference		0.494			NI	CEA (ng/ml)	≤5	Reference		0.673			NI
	Presence	1.40	0.54–3.63						>5	1.15	0.60–2.19				
Microvascular invasion	Absence	Reference		0.633			NI	HBV infection	Absence	Reference		0.210			NI
	Presence	1.17	0.61–2.23						Presence	0.27	0.04–2.09				
Lymph vessel invasion	Absence	Reference		0.287			NI	Chemotherapy	No	Reference		0.031	Reference		0.170
	Presence	1.42	0.58–1.67						Yes	0.50	0.27–0.94		0.63	0.32–1.22	

*PDAC, pancreatic ductal adenocarcinoma; HR, hazard ratio; LNR, lymph node ratio; LN, lymph node metastasis; PI, prognostic index; NLR, neutrophil-to-lymphocyte ratio; dNLR, derived neutrophil-to-lymphocyte ratio; PLR, platelet-to-lymphocyte ratio; PNI, prognostic nutritional index; SII, systemic immune-inflammation index; mGPS, modified Glasgow Prognostic Score; WBC, white blood cell; ALB, albumin; CRP, C-reactive protein; CA19-9, carbohydrate antigen 19-9; CEA, carcinoembryonic antigen; HBV, hepatitis B virus*.

### Risk Factors for Progression-Free Survival and Overall Survival

**Figure 5:**
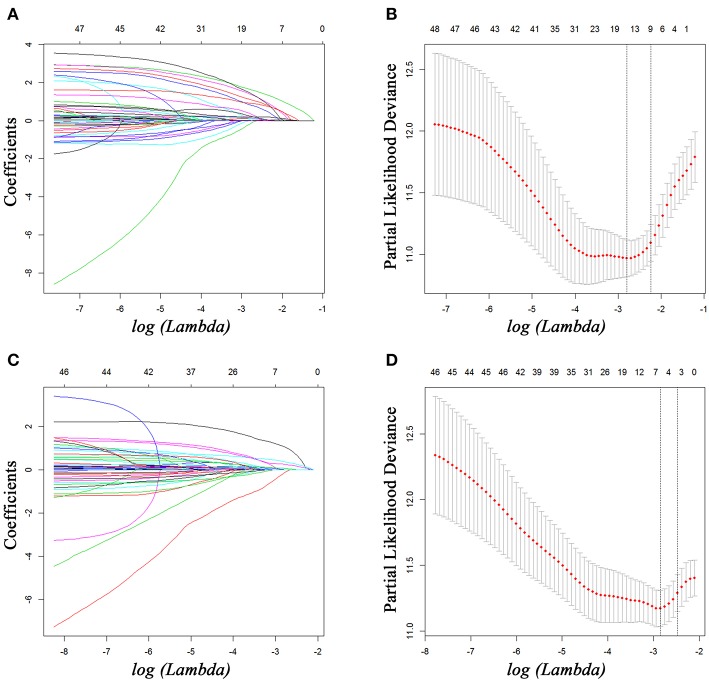
Feature selection using the least absolute shrinkage and selection operator (LASSO) Cox regression model. LASSO coefficient profiles of 48 variables against the log (Lambda) sequence for PFS **(A)** and tuning parameter (Lambda) selection in the LASSO model used 10-fold cross-validation via minimum criteria for PFS **(B)**. LASSO coefficient profiles of 48 variables against the log (Lambda) sequence for OS **(C)** and tuning parameter (Lambda) selection in the LASSO model used 10-fold cross-validation via minimum criteria for OS **(D)**. PFS, progression-free survival; OS, overall survival.

For all included patients, 1-, 2-, and 3-year OS and PFS were 81.3, 58.4, and 47.0% and 49.7, 36.0, and 29.7%, respectively. In order to investigate the prognostic factors of survival, a total of 48 high-dimensional radiological and pathological data were incorporated in the LASSO regression (Figure 5). Three best predictors for OS, including LN16 metastasis, tumor differentiation, and imaging tumor size, and another eight predictors for PFS, including the eighth edition tumor–node–metastasis (TNM) stage, liver-only metastasis, lung-only metastasis, local progression, multiple metastases, LN16 metastasis, imaging tumor size, and LNR, were identified. The predictors selected by LASSO regression, along with the associated clinical factors identified by a univariate analysis, were incorporated to the multivariable analysis. Subsequent analyses illustrated that decreased time interval to progression (HR = 4.30, 95% CI 2.57–7.20, *P* < 0.001), elevated CA19-9 level (HR = 1.92, 95% CI 1.03–3.58, *P* = 0.039), and LN16 metastasis (HR = 3.63, 95% CI 1.68–7.82, *P* = 0.001) were independent predictors for reduced OS (Table 4). Independent prognostic factors for PFS included elevated CEA level (HR = 1.78, 95% CI 1.25–2.53, *P* = 0.002), local progression (HR = 8.84, 95% CI 5.25–14.87, *P* < 0.001), liver-only metastasis (HR = 14.74, 95% CI 9.12–23.84, *P* < 0.001), lung-only metastasis (HR = 9.41, 95% CI 4.45–19.91, *P* < 0.001), local + distant progression (HR = 11.69, 95% CI 5.79–23.58, *P* < 0.001), multiple metastases (HR = 19.51, 95% CI 8.78–43.38, *P* < 0.001), LN16 metastasis (HR = 3.04, 95% CI 1.58–5.99, *P* < 0.001), imaging tumor size (HR = 1.76, 95% CI 1.16–2.67, *P* = 0.008), chemotherapy (HR = 0.60, 95% CI 0.42–0.86, *P* = 0.005), and TNM stage (HR = 2.40, 95% CI 1.17–4.92, *P* = 0.017) (Table 5).

**Table 4:** Independent prognostic factors for OS.

**Characteristics**		**Univariate analysis**			**Multivariate analysis**		
		**HR**	**95%**	***P***	**HR**	**95%**	***P***
Age	≤60 years	Reference					NI
	>60 years	1.40	0.96–2.04	0.084			
Gender	Female	Reference					NI
	Male	0.89	0.61–1.31	0.556			
WBC count	≤10	Reference			Reference		0.234
	>10	2.43	1.38–4.29	0.002	1.73	0.70–4.26	
NLR	≤3.32	Reference					NI
	>3.32	1.16	0.79–1.72	0.447			
dNLR	≤3.32	Reference					NI
	>3.32	1.03	0.69–1.54	0.903			
PLR	≤98.13	Reference					NI
	>98.13	1.16	0.65–2.08	0.612			
PNI	0	Reference					NI
	1	1.30	0.81–2.08	0.285			
SII	≤1,000	Reference					NI
	>1,000	0.93	0.61–1.40	0.712			
mGPS	0	Reference					NI
	1	1.40	0.89–2.21	0.143			
	2	1.03	0.58–1.83	0.927			
PI	0	Reference					NI
	1	1.20	0.78–1.83	0.412			
	2	2.24	1.18–4.26	0.014			
ALB (g/L)	≤35	Reference					NI
	>35	0.97	0.59–1.59	0.897			
CRP (ng/L)	≤3	Reference					NI
	>3	1.24	0.84–1.84	0.275			
CA19-9 (U/ml)	≤35	Reference			Reference		0.039
	>35	2.72	1.52–4.87		1.92	1.03–3.58	
CEA (ng/ml)	≤5	Reference			Reference		0.840
	>5	1.01	1.00–1.02	0.019	1.05	0.68–1.61	
HBV infection	Absence	Reference					NI
	Presence	1.23	0.54–2.81	0.624			
Chemotherapy	No	Reference					NI
	Yes	0.81	0.56–1.19	0.288			
Time period to recurrence (month)	≤6	Reference			Reference		
	6–12	2.45	1.34–3.57	<0.001	2.67	1.52–4.69	<0.001
	12–24	3.33	1.35–4.37	<0.001	3.29	2.00–5.43	<0.001
	>24	4.23	2.34–6.45	<0.001	4.30	2.57–7.20	<0.001
LN16 metastasis	Absence				Reference		
	Presence				3.63	1.68–7.82	0.001
Tumor differentiation	Well				Reference		
	Moderate				1.37	0.91–2.05	0.130
	Poor				1.45	0.87–2.98	0.13
Imaging tumor size (cm)	≤2				Reference		
	2–4				1.15	0.84–1.56	0.389
	>4				1.34	0.76–1.78	0.267

*OS, overall survival; HR, hazard ratio; WBC, white blood cell; NLR, neutrophil-to-lymphocyte ratio; dNLR, derived neutrophil-to-lymphocyte ratio; PLR, platelet-to-lymphocyte ratio; PNI, prognostic nutritional index; SII, systemic immune-inflammation index; mGPS, modified Glasgow Prognostic Score; PI, prognostic index; ALB, albumin; CRP, C-reactive protein; CA19-9, carbohydrate antigen 19-9; CEA, carcinoembryonic antigen; HBV, hepatitis B virus*.

**Table 5:** Independent prognostic factors for PFS.

**Characteristics**		**Univariate analysis**			**Multivariate analysis**		
		**HR**	**95%**	***P***	**HR**	**95%**	***P***
Age	≤60 years	Reference					NI
	>60 years	1.15	0.85–1.55	0.365			
Gender	Female	Reference					NI
	Male	1.15	0.85–1.56	0.375			
WBC count	≤10	Reference			Reference		0.052
	>10	1.73	1.05–2.85	0.032	1.74	0.99–3.05	
NLR	≤3.32	Reference					NI
	>3.32	1.14	0.84–1.56	0.390			
dNLR	≤3.32	Reference					NI
	>3.32	0.94	0.69–1.29	0.717			
PLR	≤98.13	Reference					NI
	>98.13	1.32	0.82–2.13	0.256			
PNI	0	Reference					NI
	1	1.25	0.86–1.82	0.244			
SII	≤1,000	Reference					NI
	>1,000	0.96	0.70–1.32	0.813			
mGPS	0	Reference					NI
	1	1.34	0.95–1.91	0.099			
	2	1.01	0.62–1.62	0.987			
PI	0	Reference					NI
	1	1.19	0.85–1.66	0.298			
	2	1.68	0.96–2.93	0.069			
ALB (g/L)	≤35	Reference					NI
	>35	1.05	0.69–1.58	0.825			
CRP (ng/L)	≤3	Reference					NI
	>3	1.22	0.89–1.66	0.216			
CA19-9 (U/ml)	≤35	Reference			Reference		0.997
	>35	1.87	1.22–2.86	0.004	0.99	0.62–1.62	
CEA (ng/ml)	≤5	Reference			Reference		0.002
	>5	1.60	1.18–2.18	0.003	1.78	1.25–2.53	
HBV infection	Absence	Reference					NI
	Presence	0.98	0.52–1.86	0.95			
Chemotherapy	No	Reference			Reference		0.005
	Yes	1.35	1.00–1.82	0.050	0.60	0.42–0.86	
Local recurrence	Absence				Reference		<0.001
	Presence				8.84	5.25–14.87	
Liver metastasis	Absence				Reference		<0.001
	Presence				14.74	9.12–23.84	
Lung metastasis	Absence				Reference		<0.001
	Presence				9.41	4.45–19.91	
Local + distant metastasis	Absence				Reference		<0.001
	Presence				11.69	5.79–23.58	
Multiple metastasis	Absence				Reference		<0.001
	Presence				19.51	8.78–43.38	
LN16 metastasis	Absence				Reference		0.001
	Presence				3.04	1.58–5.99	
Imaging tumor size (cm)	≤2				Reference		
	2–4				1.76	1.16–2.67	0.008
	>4				1.47	0.83–2.58	0.185
LNR	0				Reference		
	0–0.16				0.96	0.46–1.98	0.900
	>0.16				1.03	0.51–2.10	0.928
TNM stage	IA				Reference		
	IB				0.92	0.52–1.64	0.782
	IIA				2.40	1.17–4.92	0.017
	IIB				1.21	0.49–3.01	0.674
	III				1.12	0.50–2.50	0.789
Tumor differentiation	Well				Reference		
	Moderate				1.24	0.78–1.95	0.330
	Poor				1.65	1.21–3.67	0.032

*PFS, progression-free survival; HR, hazard ratio; WBC, white blood cell; NLR, neutrophil-to-lymphocyte ratio; dNLR, derived neutrophil-to-lymphocyte ratio; PLR, platelet-to-lymphocyte ratio; PNI, prognostic nutritional index; SII, systemic immune-inflammation index; mGPS, modified Glasgow Prognostic Score; PI, prognostic index; ALB, albumin; CRP, C-reactive protein; CA19-9, carbohydrate antigen 19-9; CEA, carcinoembryonic antigen; HBV, hepatitis B virus; LNR, lymph node ratio; TNM, tumor–node–metastasis*.

### Performance of Prediction for Overall Survival and Progression-Free Survival

**Figure 6:**
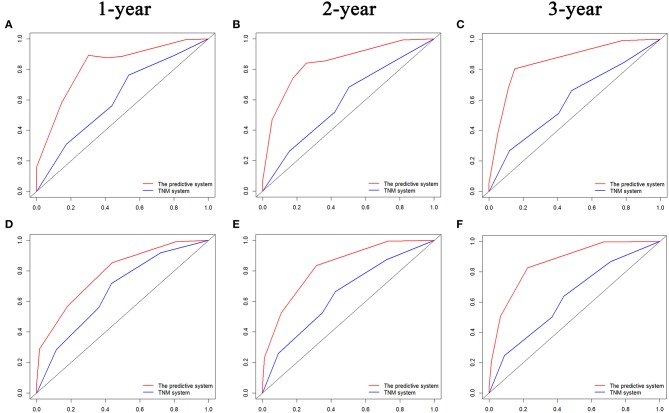
Comparisons of receiver operating characteristic (ROC) curves of both the predictive system and TNM stage system for predicting 1-, 2-, and 3-year OS **(A–C)** and PFS **(D–F)** for LAPC patients after surgery, respectively. TNM, tumor–node–metastasis; PFS, progression-free survival; OS, overall survival; LAPC, locally advanced pancreatic cancer.

The comparisons of ROC curves of the predictive systems on the basis of the risk factors and TNM stage system are shown in Figure 6. The values of AUC for 1-, 2-, and 3-year OS and PFS prediction were 0.823, 0.844, and 0.858 and 0.789, 0.829, and 0.863, respectively, which were significant higher than those of the TNM stage system (OS, 1 year, 0.614; 2 years, 0.592; 3 years, 0.599; PFS, 1 year, 0.669; 2 years, 0.647; 3 years, 0.630). The predictive system also demonstrated significantly more valuable prediction performance with the C-indexes of 0.829 (95% CI 0.760–0.898) for OS and 0.797 (95% CI 0.723–0. 871) for PFS, respectively, than did the TNM stage system (C-index, OS, 0.588 [95% CI 0.465–0.711]; PFS, 0.619 [95% CI 0.524–0.713]).

## Discussion

Pancreatic cancer has an extremely poor prognosis even after surgical resection. Recurrence was observed in more than 60% of all PDAC patients after surgery (4, 16) and remained the main reason of poor prognosis in these patients. In this study, recurrence was observed in 57.3% of patients. In addition, 68.2% of recurrences occurred at a distant site, illustrating that there were systemic diseases in these patients at the time of surgery. Also, 41.6% of recurrences occurred 2 years after surgery. Maybe recurrence-free survival for 2 years did not mean cure, and regular follow-up was also needed for these patients. Furthermore, it was shown that different time intervals or patterns of recurrence would both have different survival. These results suggested that maybe recurrence time interval and patterns were important aspects of recurrence, and the evaluation of factors associated with time intervals and patterns of recurrence opened the door to the exploration of unique biological behaviors of PDAC.

Although the prognostic value of recurrence in survival had been illustrated by previous studies, the current published results differed considerably. For instance, the recurrence rates of PDAC patients after surgery ranged from 38 to 88% in previous studies (17–21). The discrepancies might differ greatly owing to the variations of neoadjuvant treatment regimen and differences of time periods of follow-up. Additionally, the patterns and timing of recurrence of PDAC patients were also not clearly illustrated owing to small population size and limited period of follow-up (22, 23). Moreover, only “local,” “distant,” and “local + distant” groups were analyzed in most of these studies (8, 9, 24–26), and specific recurrence sites were seldom illustrated. In the present study, our detailed recurrence data allowed for further stratification of recurrence patterns in six separated groups: local, liver-only, lung-only, other, local + distant, and multiple metastases. Similar with those in other studies (27, 28), liver-only metastasis and local recurrence contributed to most of the disease progressions. Considering the time period to tumor progress, our study further illustrated that liver-only metastasis occurred mainly in early phase after surgery and diminished over time. Oppositely, other patterns of progressions, including local recurrence and lung metastasis, were more and more common along with time. Following the variations of progression patterns over time, patients might benefit from changes of therapy focus during the period of follow-up.

Progression patterns and time period were two important natural aspects of progression. Apart from the changes of progression patterns over time, it was shown that survival differences were significant when they were stratified by different sites of first recurrence and time periods to tumor progression. In the current study, liver-only metastasis led to the shortest median PFS of only 5.1 months, which was comparable with that of local + distant progression or multiple metastases. Owing to the high rates of occurrence, liver-only metastasis contributed to most of the local + distant progression and multiple metastases. This may partly explain the similar PFS among these three patterns of progression. Similar results were also observed in a study conducted by Suenaga et al. (3), which reported that the median PFS of PDAC patients after surgery was 6.0 months. Apart from liver-only and lung-only metastases, other sites of sole metastasis contributed the longest median PFS (median 12.7 months) among all patterns of progression, followed by local recurrence with a median PFS of 9.0 months. A similar result was also achieved in Vincent's study (4). Additionally, survival differences of OS and PPS were also explored in the present study. Compared with patients with liver-only metastasis, although patients with other sites of distant metastases had slightly short median PPS, they finally achieved longer median OS owing to the significantly extended PFS. Moreover, compared with other patterns of progression, local recurrence contributed to better OS, followed by other sites of sole metastasis, and better PPS, followed by lung-only metastasis. A complete understanding of why local recurrence and lung-only metastasis were associated with relatively favorable PPS remains elusive. A hypothesis assumed that the large capacity of tumor bed and lung allowed patients to endure a greater tumor burden, leading to extended survival (29). Considering the slow growth pattern and apparently less aggressive tumor biology of local progression and lung-only metastasis, maybe locally advanced pancreatic cancer (LAPC) patients can benefit from additional treatment of the subsequent lung and local recurrence after surgery. Additionally, the inherent nature of organ-specific metastasis might be explained by the distinct genetic signatures of both primary PDAC and metastatic lesions. The analysis of biological mechanisms would potentially provide personal therapeutic approaches.

The exploration of risk factors for organ-specific recurrences and predictive factors for survival formed another important finding of this study. Several characteristics were risk factors for liver-only metastasis, such as age older than 60 years and LN15 metastasis. Presence of specific stations of LN metastases could be interpreted as signs of increasing probabilities of progressions. LN18, LN15, and LN14 metastases were identified as predictors for local progression, local + distant metastases, and multiple metastases, respectively. Additionally, as an effective adjuvant therapy to increase survival, the effects of chemotherapy on patterns of progression were poorly understood. Similar with previous study (4), the current study showed that chemotherapy significantly reduced the likelihood of recurrence, especially for local recurrence and lung-only metastasis. Additionally, the prognostic factors were also explored. Apart from the conventional recurrence patterns, elevated levels of CEA, enlarged imaging tumor size, poor differentiation, and advanced TNM stages were all predictive factors of decreased PFS. The exact relation of poor differentiation and poor PFS remained unclear. Maybe this could be partly explained by the ability of PDAC to develop distant metastases, which could be enhanced by the molecules released by the poorly differentiated tumor, including epidermal growth factor, E-cadherin (24). On the other hand, an increasing time prior to tumor progression was also a predictive factor of improved OS, indicating more favorable tumor behavior in patients with late progression. After other risk factors were controlled, the multivariate analysis also illustrated that elevated level of CA19-9 and LN16 metastasis were significantly associated with decreased OS, suggesting that patients with these unfavorable characteristics needed to receive adjuvant therapy after surgery to earn prolonged survival. Similar with study conducted by Groot et al. (30), our results showed that chemotherapy was associated with less local progression and lung-only metastasis and was an independent predictor for PFS. However, the significant associations between chemotherapy and other patterns of recurrences were not observed, and chemotherapy failed to act as a predictor of OS in this study. Owing to the heterogeneity in the length and regimen of the chemotherapy, data on the adjuvant or neoadjuvant chemotherapy in the current literatures were often limited and contradictory. A previous study based on 1,375 patients did not show survival benefit from adjuvant chemotherapy (31), whereas in another study, the additional survival benefit from adjuvant chemotherapy was reported in PDAC patients (32). The selection bias partly contributed to this discrepancy in retrospective study, and maybe more insights concerning the survival benefit of chemotherapy were available from prospective studies.

It is important to note that the precise prediction of progression is essential for the individual treatment. An important advantage of this study was the use of a relatively large cohort to determine the risk factors for different patterns of recurrences and survival. Several independent prognostic factors were selected by evaluating high-dimensional radiological and clinicopathological variables in the current study. In addition, analyses of ROC curves and comparisons of the associated values of AUC and C-indexes of the predictive system and TNM stage system showed a strong predictive strength of the predictive system on the basis of risk factors for OS and PFS. The inclusion of additional clinicopathological variables guaranteed that the established predictive system was better in predicting OS and PFS than did the eighth edition of the TNM stage system. On the other hand, the different clinicopathological features of progression patterns and timing suggested that there might be unique biological features in different progressions. Currently, the molecular feature, *SMAD4*, was shown to have a close relationship with progression patterns. Tumors with *SMAD4* up-regulated tended to be localized, whereas the down-regulation or silence of this gene was likely to promote metastasis (33). Moreover, different regulation of specific genes was associated closely with different patterns of progressions in an animal model (34, 35). Therefore, maybe the combination of clinicopathological characteristics and genetic features would have more meaningful implications in predicting progressions. Clinicians could perform evaluation of recurrence risks and survival on the basis of individual risk factors of patients and specialize the adjuvant therapy, which fitted the current trend to personalized medicine.

This study has several limitations. First, the specific adjuvant therapies after surgery and the associated response to adjuvant therapy were unavailable. More detailed information of length and regimen of chemotherapy would further illustrate the association between therapy and progression. Second, this study only focused on the first recurrence, and subsequent progressions were not taken into accounted. Third, it was well-known that more progressions would be observed over time. In this study, the period of follow-up for all included patients was longer than 1 year, but this time period was not relatively long enough. Although patients were followed up with a median time of 2 years in this study, the whole view of progression in patients could be changed if patients were followed up even longer. A prospective study with an even longer period of follow-up is also needed to validate results of this study. Last, sometimes diagnoses of progression on the basis of imaging were challenging, and it was possible to overestimate the probabilities of progression in PDAC patients after surgery.

In conclusion, for PDAC patients after radical operation, the different patterns and timing of recurrence were accurately described in the present study. This study further identified the risk factors of different recurrence patterns, which could help to predict the occurrence of first tumor progression. Furthermore, individual predictors of OS and PFS were also identified and validated for these patients. These findings further suggested the linkages between different progression patterns and biological heterogeneity, and the exploration might provide new versions into the prediction of tumor progression, prognosis stratification, and a more personalized management for PDAC patients after surgery.

## Data Availability Statement

The authenticity of this article has been validated by uploading the key raw data onto the Research Data Deposit public platform (http://www.researchdata.org.cn), with the Approval Number as RDDA2019001267.

## Ethics Statement

This study was approved by the Institutional Review Board of SYSUCC. All procedures performed in present study involving human participants were in accordance with the ethical standards of institutional and/or national research committees and the 1964 Declaration of Helsinki and its later amendments or similar ethical standards. Written informed consent for inclusion in this study was obtained from patients prior to treatment.

## Author Contributions

SL was responsible for conception, design, and quality control of this study. CH, XH, and YZ performed the study selection, data extraction, statistical analyses and were major contributors in writing the manuscript. CH and XH participated in study selection and statistical analyses. CH, XH, YZ, ZC, and XL contributed in classification criteria discussion. CH, XH, and YZ contributed to the writing of manuscript. SL reviewed and edited the manuscript. All authors have read and approved the final version of the manuscript.

### Conflict of Interest

The authors declare that the research was conducted in the absence of any commercial or financial relationships that could be construed as a potential conflict of interest.

## References

[1] Rahib L Smith BD Aizenberg R Rosenzweig AB Fleshman JM Matrisian LM. Projecting cancer incidence and deaths to 2030: the unexpected burden of thyroid, liver, and pancreas cancers in the United States. Cancer Res. (2014) 74:2913–21. 10.1158/0008-5472.CAN-14-0155 24840647

[2] Parikh AA Maiga A Bentrem D Squires MH III Kooby DA Maithel SK. Adjuvant therapy in pancreas cancer: does it influence patterns of recurrence? J Am Coll Surg. (2016) 222:448–56. 10.1016/j.jamcollsurg.2015.12.031 26895735 PMC10191770

[3] Suenaga M Fujii T Kanda M Takami H Okumura N Inokawa Y. Pattern of first recurrent lesions in pancreatic cancer: hepatic relapse is associated with dismal prognosis and portal vein invasion. Hepato Gastroenterol. (2014) 61:1756–61. 10.5754/hge13637 25436375

[4] Groot VP Rezaee N Wu W Cameron JL Fishman EK Hruban RH. Patterns, timing, and predictors of recurrence following pancreatectomy for pancreatic ductal adenocarcinoma. Ann Surg. (2018) 267:936–45. 10.1097/SLA.0000000000002234 28338509

[5] Ellison LF Wilkins K. An update on cancer survival. Health Rep. (2010) 21:55–60. 20973434

[6] Tummers WS Groen JV Sibinga Mulder BG Farina-Sarasqueta A Morreau J Putter H. Impact of resection margin status on recurrence and survival in pancreatic cancer surgery. Br J Surg. (2019) 106:1055–65. 10.1002/bjs.11115 30883699 PMC6617755

[7] Kim NH Kim HJ. Preoperative risk factors for early recurrence in patients with resectable pancreatic ductal adenocarcinoma after curative intent surgical resection. Hepatobiliary Pancreat Dis Int. (2018) 17:450–5. 10.1016/j.hbpd.2018.09.003 30237091

[8] Van den Broeck A Sergeant G Ectors N Van Steenbergen W Aerts R Topal B. Patterns of recurrence after curative resection of pancreatic ductal adenocarcinoma. Eur J Surg Oncol. (2009) 35:600–4. 10.1016/j.ejso.2008.12.006 19131205

[9] Sperti C Pasquali C Piccoli A Pedrazzoli S. Recurrence after resection for ductal adenocarcinoma of the pancreas. World J Surg. (1997) 21:195–200. 10.1007/s002689900215 8995078

[10] Chang DK Johns AL Merrett ND Gill AJ Colvin EK Scarlett CJ. Margin clearance and outcome in resected pancreatic cancer. J Clin Oncol. (2009) 27:2855–62. 10.1200/JCO.2008.20.5104 19398572

[11] Gebauer F Tachezy M Vashist YK Marx AH Yekebas E Izbicki JR Resection margin clearance in pancreatic cancer after implementation of the Leeds Pathology Protocol (LEEPP): clinically relevant or just academic? World J Surg. (2015) 39:493–9. 10.1007/s00268-014-2808-4 25270344

[12] Tempero MA Malafa MP Al-Hawary M Asbun H Bain A Behrman SW. Pancreatic adenocarcinoma, version 2.2017, NCCN clinical practice guidelines in oncology. J Natl Compr Cancer Netw. (2017) 15:1028–61. 10.6004/jnccn.2017.0131 28784865

[13] Amin MB ES Greene F. AJCC Cancer Staging Manual. 8th ed. Chicago, IL: Springer (2017).

[14] He CB Lin XJ. Inflammation scores predict the survival of patients with hepatocellular carcinoma who were treated with transarterial chemoembolization and recombinant human type-5 adenovirus H101. PLoS ONE. (2017) 12:e0174769. 10.1371/journal.pone.0174769 28355305 PMC5371390

[15] De Giorgi U Procopio G Giannarelli D Sabbatini R Bearz A Buti S. Association of systemic inflammation index and body mass index with survival in patients with renal cell cancer treated with nivolumab. Clin Cancer Res. (2019) 25:3839–46. 10.1158/1078-0432.CCR-18-3661 30967420

[16] Kim YI Song KB Lee YJ Park KM Hwang DW Lee JH. Management of isolated recurrence after surgery for pancreatic adenocarcinoma. Br J Surg. (2019) 106:898–909. 10.1002/bjs.11144 31162655

[17] Katz MH Pisters PW Evans DB Sun CC Lee JE Fleming JB. Borderline resectable pancreatic cancer: the importance of this emerging stage of disease. J Am Coll Surg. (2008) 206:833–46; discussion: 46–8. 10.1016/j.jamcollsurg.2007.12.020 18471707 PMC5901743

[18] Kadera BE Sunjaya DB Isacoff WH Li L Hines OJ Tomlinson JS. Locally advanced pancreatic cancer: association between prolonged preoperative treatment and lymph-node negativity and overall survival. JAMA Surg. (2014) 149:145–53. 10.1001/jamasurg.2013.2690 24306217

[19] Ferrone CR Marchegiani G Hong TS Ryan DP Deshpande V McDonnell EI. Radiological and surgical implications of neoadjuvant treatment with FOLFIRINOX for locally advanced and borderline resectable pancreatic cancer. Ann Surg. (2015) 261:12–7. 10.1097/SLA.0000000000000867 25599322 PMC4349683

[20] Kim HS Jang JY Han Y Lee KB Joo I Lee DH. Survival outcome and prognostic factors of neoadjuvant treatment followed by resection for borderline resectable pancreatic cancer. Ann Surg Treat Res. (2017) 93:186–94. 10.4174/astr.2017.93.4.186 29094028 PMC5658300

[21] Jang JY Han Y Lee H Kim SW Kwon W Lee KH. Oncological benefits of neoadjuvant chemoradiation with gemcitabine versus upfront surgery in patients with borderline resectable pancreatic cancer: a prospective, randomized, open-label, multicenter phase 2/3 trial. Ann Surg. (2018) 268:215–22. 10.1097/SLA.0000000000002705 29462005

[22] Zhao Y Wang C. Clinicopathological features, recurrence patterns, and prognosis of pancreatic adenocarcinoma with normal serum CA19–9. A consecutive series of 154 cases from a single institute. J Gastrointest Surg. (2019). 10.1007/s11605-019-04209-w. [Epub ahead of print]. 30945087

[23] Li S Xu H Wang W Gao H Li H Zhang S. The systemic inflammation response index predicts survival and recurrence in patients with resectable pancreatic ductal adenocarcinoma. Cancer Manage Res. (2019) 11:3327–37. 10.2147/CMAR.S197911 31114368 PMC6489619

[24] Shibata K Matsumoto T Yada K Sasaki A Ohta M Kitano S. Factors predicting recurrence after resection of pancreatic ductal carcinoma. Pancreas. (2005) 31:69–73. 10.1097/01.mpa.0000166998.04266.88 15968250

[25] Zhang Y Frampton AE Kyriakides C Bong JJ Habib N Ahmad R. Loco-recurrence after resection for ductal adenocarcinoma of the pancreas: predictors and implications for adjuvant chemoradiotherapy. J Cancer Res Clin Oncol. (2012) 138:1063–71. 10.1007/s00432-012-1165-7 22392075 PMC11824181

[26] Asiyanbola B Gleisner A Herman JM Choti MA Wolfgang CL Swartz M. Determining pattern of recurrence following pancreaticoduodenectomy and adjuvant 5-flurouracil-based chemoradiation therapy: effect of number of metastatic lymph nodes and lymph node ratio. J Gastrointest Surg. (2009) 13:752–9. 10.1007/s11605-008-0762-x 19089517

[27] Tani M Kawai M Miyazawa M Hirono S Ina S Nishioka R. Liver metastasis as an initial recurrence has no impact on the survival of patients with resectable pancreatic adenocarcinoma. Langenbeck's Arch Surg. (2009) 394:249–53. 10.1007/s00423-008-0296-4 18343944

[28] Hernandez JM Morton CA Al-Saadi S Villadolid D Cooper J Bowers C. The natural history of resected pancreatic cancer without adjuvant chemotherapy. Am Surg. (2010) 76:480–5. 20506876

[29] Zheng B Ohuchida K Yan Z Okumura T Ohtsuka T Nakamura M. Primary recurrence in the lung is related to favorable prognosis in patients with pancreatic cancer and postoperative recurrence. World J Surg. (2017) 41:2858–66. 10.1007/s00268-017-4068-6 28634843

[30] Groot VP Blair AB Gemenetzis G Ding D Burkhart RA Yu J. Recurrence after neoadjuvant therapy and resection of borderline resectable and locally advanced pancreatic cancer. Eur J Surg Oncol. (2019) 45:1674–83. 10.1016/j.ejso.2019.04.007 31023560

[31] de Geus SWL Kasumova GG Eskander MF Ng SC Kent TS James Moser A. Is neoadjuvant therapy sufficient in resected pancreatic cancer patients? A national study. J Gastrointest Surg. (2018) 22:214–25. 10.1007/s11605-017-3541-8 29235000

[32] Roland CL Katz MH Tzeng CW Lin H Varadhachary GR Shroff R. The addition of postoperative chemotherapy is associated with improved survival in patients with pancreatic cancer treated with preoperative therapy. Ann Surg Oncol. (2015) 22(Suppl 3):S1221–8. 10.1245/s10434-015-4854-z 26350371 PMC5192562

[33] Iacobuzio-Donahue CA Fu B Yachida S Luo M Abe H Henderson CM. DPC4 gene status of the primary carcinoma correlates with patterns of failure in patients with pancreatic cancer. J Clin Oncol. (2009) 27:1806–13. 10.1200/JCO.2008.17.7188 19273710 PMC2668706

[34] Morales M Arenas EJ Urosevic J Guiu M Fernández E Planet E. RARRES3 suppresses breast cancer lung metastasis by regulating adhesion and differentiation. EMBO Mol Med. (2014) 6:865–81. 10.15252/emmm.201303675 24867881 PMC4119352

[35] Valiente M Obenauf AC Jin X Chen Q Zhang XH Lee DJ. Serpins promote cancer cell survival and vascular co-option in brain metastasis. Cell. (2014) 156:1002–16. 10.1016/j.cell.2014.01.040 24581498 PMC3988473

