# Supplementary material for: A Quantitative Clinicopathological Signature for Predicting Recurrence Risk of Pancreatic Ductal Adenocarcinoma After Radical Resection
Source: Front Oncol. 2019 Nov 12;9:1197. doi: 10.3389/fonc.2019.01197 (PMC6861378; doi:10.3389/fonc.2019.01197)

Supplementary Material

Supplement Figure 1. Pairwise comparison of cumulative recurrence rate for different tumor progression patterns

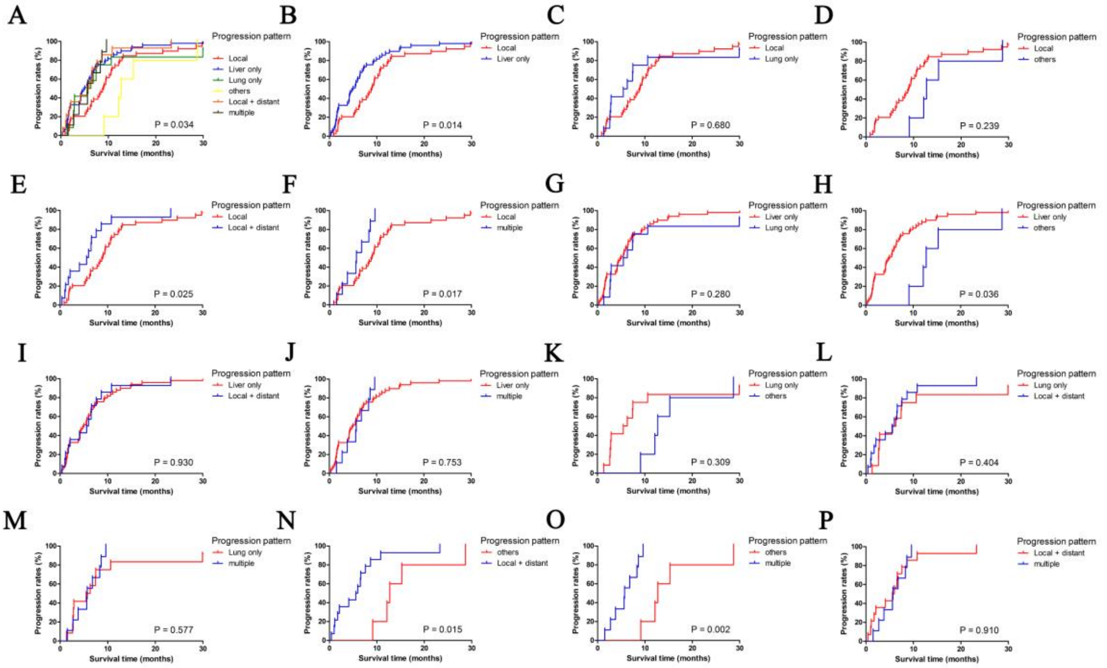

Supplement Figure 2. Pairwise comparison of post progression survival for different tumor progression patterns

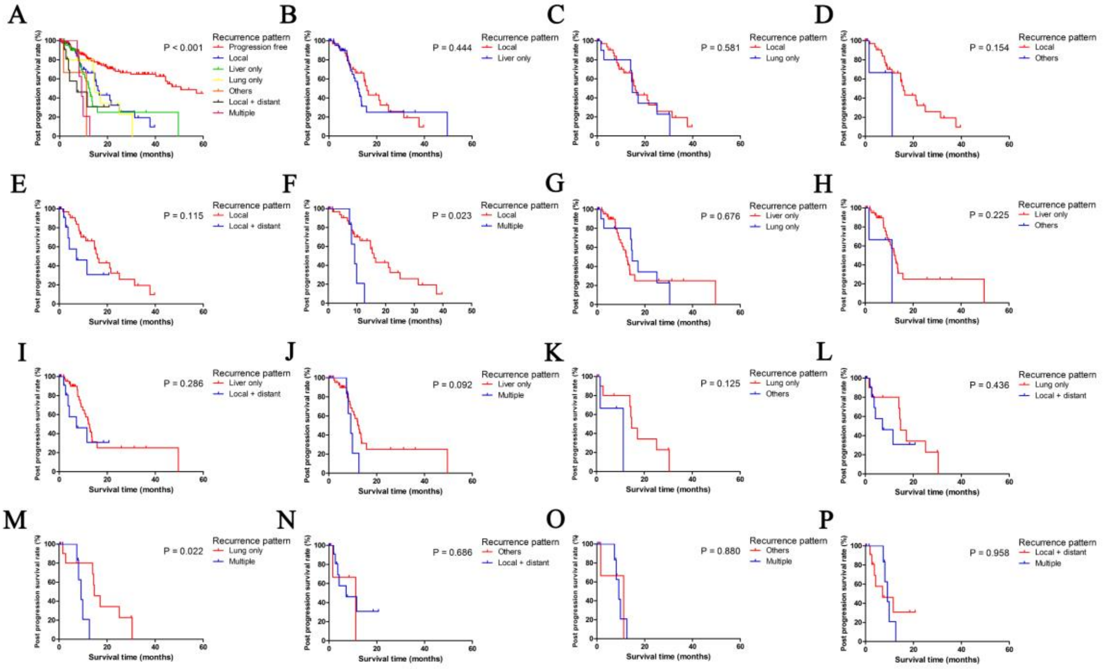

Supplement: Supplementary file 1 [file Data_Sheet_1.pdf]
