# Supplementary material for: A Quantitative Clinicopathological Signature for Predicting Recurrence Risk of Pancreatic Ductal Adenocarcinoma After Radical Resection
Source: Front Oncol. 2019 Nov 12;9:1197. doi: 10.3389/fonc.2019.01197 (PMC6861378; doi:10.3389/fonc.2019.01197)
Supplement: Supplementary file 2 [file Table_1.docx]

Supplement Table 1. Risk factors for lung metastasis in PDAC patients after surgery

| Characteristics | | Univariate analysis | | | Multivariate analysis | | | Characteristics | | Univariate analysis | | | Multivariate analysis | | |
| --- | --- | --- | --- | --- | --- | --- | --- | --- | --- | --- | --- | --- | --- | --- | --- |
|  |  | HR | 95% | P | HR | 95% | P |  |  | HR | 95% | P | HR | 95% | P |
| Age | ≤ 60 years | Reference | | 0.775 |  |  | NI | Perineural invasion | Absence | Reference | | 0.483 |  |  | NI |
|  | > 60 years | 0.84 | 0.26-4.47 |  |  |  |  |  | Presence | 0.66 | 0.20-3.12 |  |  |  |  |
| Gender | Female | Reference | | 0.661 |  |  | NI | Adjacent organ invasion | Absence | Reference | | 0.796 |  |  | NI |
|  | Male | 1.31 | 0.39-4.47 |  |  |  |  |  | Presence | 0.76 | 0.10-6.08 |  |  |  |  |
| LN metastasis | Absence | Reference | | 0.262 |  |  | NI | LNR | 0 | Reference | |  |  |  | NI |
|  | Presence | 1.96 | 0.61-6.31 |  |  |  |  |  | 0 - 0.16 | 3.05 | 0.90-10.34 | 0.074 |  |  |  |
| LN5 metastasis | Absence | Reference | |  |  |  | NI |  | > 0.16 | 1.80 | 0.89-3.64 | 0.101 |  |  |  |
|  | Presence | - |  |  |  |  |  | Satellite foci | Absence | Reference | |  |  |  | NI |
| LN6 metastasis | Absence | Reference | |  |  |  | NI |  | Presence | - |  |  |  |  |  |
|  | Presence | - |  |  |  |  |  | Tumor site | Head | Reference | | 0.166 |  |  | NI |
| LN7 metastasis | Absence | Reference | | 0.148 |  |  | NI |  | Body and tail | 2.40 | 0.70-8.28 |  |  |  |  |
|  | Presence | 5.18 | 0.5-48.19 |  |  |  |  | Imaging tumor size (cm) | ≤ 2 | Reference | |  | Reference | |  |
| LN8 metastasis | Absence | Reference | | 0.242 |  |  | NI |  | 2 – 4 | 1.04 | 0.27-4.04 | 0.956 | 4.15 | 0.40-43.44 | 0.235 |
|  | Presence | 3.68 | 0.42-32.52 |  |  |  |  |  | > 4 | 0.35 | 0.04-2.87 | 0.330 | 6.42 | 0.59-70.43 | 0.128 |
| LN9 metastasis | Absence | Reference | | 0.342 |  |  | NI | Imaging LN metastasis | Absence | Reference | | 0.571 |  |  | NI |
|  | Presence | 2.84 | 0.33-24.42 |  |  |  |  |  | Presence | 1.40 | 0.440-4.44 |  |  |  |  |
| LN10 metastasis | Absence | Reference | | 0.007 | Reference | | 0.011 | Imaging vascular invasion | Absence | Reference | | 0.849 |  |  | NI |
|  | Presence | 11.4 | 1.97-66.06 |  | 15.96 | 1.89-134.86 |  |  | Presence | 1.14 | 0.30-4.33 |  |  |  |  |
| LN11 metastasis | Absence | Reference | |  |  |  | NI | Imaging LN size (cm) | ≤ 0.5 | Reference | |  |  |  | NI |
|  | Presence | - |  |  |  |  |  |  | 0.5-1 | 1.04 | 0.27-4.04 | 0.956 |  |  |  |
| LN12 metastasis | Absence | Reference | | 0.745 |  |  | NI |  | > 1 | 0.35 | 0.04-2.87 | 0.330 |  |  |  |
|  | Presence | 0.71 | 0.09-5.66 |  |  |  |  | PI | 0 | Reference | |  |  |  | NI |
| LN13 metastasis | Absence | Reference | | 0.141 |  |  | NI |  | 1 | 0.52 | 0.11-2.44 | 0.402 |  |  |  |
|  | Presence | 2.42 | 0.75-7.89 |  |  |  |  |  | 2 | 1.17 | 0.14-9.79 | 0.883 |  |  |  |
| LN14 metastasis | Absence | Reference | | 0.196 |  |  | NI | NLR | ≤ 3.32 | Reference | | 0.47 |  |  | NI |
|  | Presence | 2.85 | 0.58-13.96 |  |  |  |  |  | > 3.32 | 0.61 | 0.16-2.32 |  |  |  |  |
| LN15 metastasis | Absence | Reference | |  |  |  |  | dNLR | ≤ 3.32 | Reference | | 0.21 |  |  | NI |
|  | Presence | - |  |  |  |  |  |  | > 3.32 | 0.48 | 0.15-1.53 |  |  |  |  |
| LN16 metastasis | Absence | Reference | | 0.725 |  |  |  | PLR | ≤ 98.13 | Reference | | 0.70 |  |  | NI |
|  | Presence | 1.46 | 0.18-11.98 |  |  |  |  |  | > 98.13 | 1.51 | 0.19-12.05 |  |  |  |  |
| LN17 metastasis | Absence | Reference | | 0.292 |  |  | NI | PNI | 0 | Reference | | 0.281 |  |  | NI |
|  | Presence | 3.21 | 0.37-27.91 |  |  |  |  |  | 1 | 3.12 | 0.40-24.58 |  |  |  |  |
| LN18 metastasis | Absence | Reference | |  |  |  | NI | SII | ≤ 1000 | Reference | | 0.608 |  |  | NI |
|  | Presence | - |  |  |  |  |  |  | > 1000 | 0.71 | 0.19-2.67 |  |  |  |  |
| Positive LN number | 0 | Reference | |  |  |  | NI | mGPS | 0 | Reference | |  |  |  | NI |
|  | 1 - 3 | 1.63 | 0.40-6.66 | 0.499 |  |  |  |  | 1 | 0.66 | 0.14-3.13 | 0.601 |  |  |  |
|  | > 3 | 3.58 | 0.76-16.81 | 0.106 |  |  |  |  | 2 | 0.67 | 0.08-5.47 | 0.709 |  |  |  |
| Pancreatic membrane invasion | Absence | Reference | | 0.678 |  |  | NI | WBC | ≤ 10 | Reference | | 0.887 |  |  | NI |
|  | Presence | 0.77 | 0.23-2.62 |  |  |  |  |  | > 10 | 1.17 | 0.14-9.46 |  |  |  |  |
| Tumor size (cm) | ≤ 2 | Reference | |  |  |  |  | ALB (g/L) | ≤ 35 | Reference | | 0.506 |  |  | NI |
|  | 2 – 4 | 5.04 | 0.62-41.03 | 0.130 |  |  |  |  | > 35 | 2.02 | 0.26-16.04 |  |  |  |  |
|  | > 4 | 4.02 | 0.41-39.49 | 0.233 |  |  |  | CRP (ng/L) | ≤ 3 | Reference | | 0.545 |  |  | NI |
| Tumor differentiation | Well | Reference | |  | Reference | |  |  | > 3 | 0.66 | 0.18-2.51 |  |  |  |  |
|  | Moderate | 0.04 | 0.01-0.73 | 0.030 | 0.05 | 0.01-1.37 | 0.075 | CA19-9 (U/ml) | ≤ 35 | Reference | | 0.798 |  |  | NI |
|  | Poor | 0.04 | 0.01-0.65 | 0.024 | 0.04 | 0.01-1.10 | 0.056 |  | > 35 | 1.22 | 0.26-5.74 |  |  |  |  |
| Macrovascular invasion | Absence | Reference | | 0.879 |  |  | NI | CEA (ng/ml) | ≤ 5 | Reference | | 0.473 |  |  | NI |
|  | Presence | 0.85 | 0.11-6.84 |  |  |  |  |  | > 5 | 1.54 | 0.48-4.97 |  |  |  |  |
| Microvascular invasion | Absence | Reference | | 0.111 |  |  | NI | HBV infection | Absence | Reference | |  |  |  | NI |
|  | Presence | 0.19 | 0.02-1.47 |  |  |  |  |  | Presence | - |  |  |  |  |  |
| Lymph vessel invasion | Absence | Reference | | 0.568 |  |  | NI | Chemotherapy | No | Reference | | 0.022 | Reference | | 0.031 |
|  | Presence | 0.57 | 0.15-4.21 |  |  |  |  |  | Yes | 0.17 | 0.04-0.78 |  | 0.14 | 0.02-0.83 |  |

LN, lymph node metastasis; LNR, lymph node ratio; PI, prognostic index; NLR, neutrophil-to-lymphocyte ratio; PLR, platelet-to-lymphocyte ratio; PNI, prognostic nutritional index; SII, systemic immune-inflammation index; mGPS, modified Glasgow Prognostic Score; WBC, white blood cell count; ALB, albumin; CRP, C-reactive protein; CA19-9, carbohydrate antigen 19-9; CEA, carcinoembryonic antigen; HBV, hepatitis B virus

Supplement Table 2. Risk factors for other metastases in PDAC patients after surgery

| Characteristics | | Univariate analysis | | | Multivariate analysis | | | Characteristics | | Univariate analysis | | | Multivariate analysis | | |
| --- | --- | --- | --- | --- | --- | --- | --- | --- | --- | --- | --- | --- | --- | --- | --- |
|  |  | HR | 95% | P | HR | 95% | P |  |  | HR | 95% | P | HR | 95% | P |
| Age | ≤ 60 years | Reference | | 0.797 |  |  | NI | Perineural invasion | Absence | Reference | | 0.708 |  |  | NI |
|  | > 60 years | 0.79 | 0.13-4.79 |  |  |  |  |  | Presence | 1.41 | 0.23-8.57 |  |  |  |  |
| Gender | Female | Reference | | 0.36 |  |  | NI | Adjacent organ invasion | Absence | Reference | | 0.056 |  |  | NI |
|  | Male | 0.43 | 0.07-2.60 |  |  |  |  |  | Presence | 5.93 | 0.95-36.94 |  |  |  |  |
| LN metastasis | Absence | Reference | | 0.330 |  |  | NI | LNR | 0 | Reference | |  |  |  | NI |
|  | Presence | 0.34 | 0.04-3.03 |  |  |  |  |  | 0 - 0.16 | - |  |  |  |  |  |
| LN5 metastasis | Absence | Reference | |  |  |  | NI |  | > 0.16 | 0.74 | 0.11-4.87 | 0.752 |  |  |  |
|  | Presence | - |  |  |  |  |  | Satellite foci | Absence | Reference | |  |  |  | NI |
| LN6 metastasis | Absence | Reference | |  |  |  | NI |  | Presence | - |  |  |  |  |  |
|  | Presence | - |  |  |  |  |  | Tumor site | Head | Reference | | 0.033 | Reference | | 0.148 |
| LN7 metastasis | Absence | Reference | |  |  |  | NI |  | Body and tail | 7.24 | 1.18-44.41 |  | 4.15 | 0.60-28.54 |  |
|  | Presence | - |  |  |  |  |  | Imaging tumor size (cm) | ≤ 2 | Reference | |  |  |  | NI |
| LN8 metastasis | Absence | Reference | |  |  |  | NI |  | 2 – 4 | - |  |  |  |  |  |
|  | Presence | - |  |  |  |  |  |  | > 4 | - |  |  |  |  |  |
| LN9 metastasis | Absence | Reference | |  |  |  | NI | Imaging LN metastasis | Absence | Reference | |  |  |  | NI |
|  | Presence | - |  |  |  |  |  |  | Presence | - |  |  |  |  |  |
| LN10 metastasis | Absence | Reference | |  |  |  | NI | Imaging vascular invasion | Absence | Reference | | 0.883 |  |  | NI |
|  | Presence | - |  |  |  |  |  |  | Presence | 0.85 | 0.09-7.71 |  |  |  |  |
| LN11 metastasis | Absence | Reference | |  |  |  | NI | Imaging LN size (cm) | ≤ 0.5 | Reference | |  |  |  | NI |
|  | Presence | - |  |  |  |  |  |  | 0.5-1 | 0.92 | 0.09-9.01 | 0.943 |  |  |  |
| LN12 metastasis | Absence | Reference | |  |  |  | NI |  | > 1 | 0.97 | 0.10-9.47 | 0.977 |  |  |  |
|  | Presence | - |  |  |  |  |  | PI | 0 | Reference | |  |  |  | NI |
| LN13 metastasis | Absence | Reference | | 0.852 |  |  | NI |  | 1 | 1.59 | 0.26-9.71 | 0.613 |  |  |  |
|  | Presence | 0.81 | 0.09-7.37 |  |  |  |  |  | 2 | - |  |  |  |  |  |
| LN14 metastasis | Absence | Reference | |  |  |  | NI | NLR | ≤ 3.32 | Reference | | 0.495 |  |  | NI |
|  | Presence | - |  |  |  |  |  |  | > 3.32 | 0.46 | 0.05-4.21 |  |  |  |  |
| LN15 metastasis | Absence | Reference | |  |  |  | NI | dNLR | ≤ 3.32 | Reference | | 0.220 |  |  | NI |
|  | Presence | - |  |  |  |  |  |  | > 3.32 | 0.32 | 0.05-1.97 |  |  |  |  |
| LN16 metastasis | Absence | Reference | |  |  |  | NI | PLR | ≤ 98.13 | Reference | | 0.008 | Reference | | 0.036 |
|  | Presence | - |  |  |  |  |  |  | > 98.13 | 0.08 | 0.01-0.52 |  | 0.13 | 0.02-0.87 |  |
| LN17 metastasis | Absence | Reference | |  |  |  | NI | PNI | 0 | Reference | | 0.326 |  |  | NI |
|  | Presence | - |  |  |  |  |  |  | 1 | 0.40 | 0.07-2.47 |  |  |  |  |
| LN18 metastasis | Absence | Reference | |  |  |  | NI | SII | ≤ 1000 | Reference | |  |  |  | NI |
|  | Presence | - |  |  |  |  |  |  | > 1000 | - |  |  |  |  |  |
| Positive LN number | 0 | Reference | | 0.739 |  |  | NI | mGPS | 0 | Reference | |  |  |  | NI |
|  | 1 - 3 | 1.61 | 0.10-26.00 |  |  |  |  |  | 1 | - |  |  |  |  |  |
|  | > 3 | - |  |  |  |  |  |  | 2 | 2.07 | 0.21-20.55 | 0.533 |  |  |  |
| Pancreatic membrane invasion | Absence | Reference | | 0.395 |  |  | NI | WBC | ≤ 10 | Reference | |  |  |  | NI |
|  | Presence | 0.39 | 0.04-3.48 |  |  |  |  |  | > 10 | - |  |  |  |  |  |
| Tumor size (cm) | ≤ 2 | Reference | |  |  |  | NI | ALB (g/L) | ≤ 35 | Reference | | 0.147 |  |  | NI |
|  | 2 – 4 | 0.30 | 0.03-3.32 | 0.324 |  |  |  |  | > 35 | 0.26 | 0.04-1.61 |  |  |  |  |
|  | > 4 | 1.30 | 0.18-9.50 | 0.794 |  |  |  | CRP (ng/L) | ≤ 3 | Reference | | 0.742 |  |  | NI |
| Tumor differentiation | Well | Reference | |  |  |  | NI |  | > 3 | 1.35 | 0.22-8.24 |  |  |  |  |
|  | Moderate | - |  |  |  |  |  | CA19-9 (U/ml) | ≤ 35 | Reference | | 0.264 |  |  | NI |
|  | Poor | - |  |  |  |  |  |  | > 35 | 0.36 | 0.06-2.18 |  |  |  |  |
| Macrovascular invasion | Absence | Reference | |  |  |  | NI | CEA (ng/ml) | ≤ 5 | Reference | | 0.202 |  |  | NI |
|  | Presence | - |  |  |  |  |  |  | > 5 | 3.24 | 0.53-19.71 |  |  |  |  |
| Microvascular invasion | Absence | Reference | |  |  |  | NI | HBV infection | Absence | Reference | |  |  |  | NI |
|  | Presence | - |  |  |  |  |  |  | Presence | - |  |  |  |  |  |
| Lymph vessel invasion | Absence | Reference | | 0.578 |  |  | NI | Chemotherapy | No | Reference | | 0.562 |  |  | NI |
|  | Presence | 1.57 | 0.57-5.74 |  |  |  |  |  | Yes | 0.59 | 0.10-3.56 |  |  |  |  |

Abbreviations as in Supplement Table 1

Supplement Table 3. Risk factors for local + distant metastases in PDAC patients after surgery

| Characteristics | | Univariate analysis | | | Multivariate analysis | | | Characteristics | | Univariate analysis | | | Multivariate analysis | | |
| --- | --- | --- | --- | --- | --- | --- | --- | --- | --- | --- | --- | --- | --- | --- | --- |
|  |  | HR | 95% | P | HR | 95% | P |  |  | HR | 95% | P | HR | 95% | P |
| Age | ≤ 60 years | Reference | | 0.827 |  |  | NI | Perineural invasion | Absence | Reference | | 0.899 |  |  | NI |
|  | > 60 years | 0.89 | 0.30-2.62 |  |  |  |  |  | Presence | 0.93 | 0.32-2.73 |  |  |  |  |
| Gender | Female | Reference | | 0.787 |  |  | NI | Adjacent organ invasion | Absence | Reference | | 0.67 |  |  | NI |
|  | Male | 0.86 | 0.29-2.55 |  |  |  |  |  | Presence | 0.64 | 0.08-5.04 |  |  |  |  |
| LN metastasis | Absence | Reference | | 0.10 |  |  | NI | LNR | 0 | Reference | |  |  |  | NI |
|  | Presence | 2.56 | 0.84-7.82 |  |  |  |  |  | 0 - 0.16 | 2.26 | 0.71-7.18 | 0.166 |  |  |  |
| LN5 metastasis | Absence | Reference | |  |  |  | NI |  | > 0.16 | 1.78 | 0.93-3.40 | 0.084 |  |  |  |
|  | Presence | - |  |  |  |  |  | Satellite foci | Absence | Reference | | 0.703 |  |  | NI |
| LN6 metastasis | Absence | Reference | |  |  |  | NI |  | Presence | 1.51 | 0.18-12.34 |  |  |  |  |
|  | Presence | - |  |  |  |  |  | Tumor site | Head | Reference | | 0.723 |  |  | NI |
| LN7 metastasis | Absence | Reference | |  |  |  | NI |  | Body and tail | 1.27 | 0.34-4.71 |  |  |  |  |
|  | Presence | - |  |  |  |  |  | Imaging tumor size (cm) | ≤ 2 | Reference | |  |  |  | NI |
| LN8 metastasis | Absence | Reference | |  |  |  | NI |  | 2 – 4 | 2.30 | 0.61-8.70 | 0.22 |  |  |  |
|  | Presence | - |  |  |  |  |  |  | > 4 | 1.22 | 0.20-7.55 | 0.827 |  |  |  |
| LN9 metastasis | Absence | Reference | | 0.426 |  |  | NI | Imaging LN metastasis | Absence | Reference | | 0.539 |  |  | NI |
|  | Presence | 2.39 | 0.28-20.26 |  |  |  |  |  | Presence | 1.40 | 0.48-4.10 |  |  |  |  |
| LN10 metastasis | Absence | Reference | | 0.011 | Reference | | 0.204 | Imaging vascular invasion | Absence | Reference | | 0.446 |  |  | NI |
|  | Presence | 9.43 | 1.66-53.67 |  | 6.24 | 0.37-105.17 |  |  | Presence | 0.55 | 0.12-2.53 |  |  |  |  |
| LN11 metastasis | Absence | Reference | | 0.018 | Reference | | 0.57 | Imaging LN size (cm) | ≤ 0.5 | Reference | |  | Reference | |  |
|  | Presence | 7.83 | 1.42-42.94 |  | 2.26 | 0.14-36.22 |  |  | 0.5-1 | 4.23 | 1.29-13.83 | 0.017 | 4.57 | 1.34-15.60 | 0.015 |
| LN12 metastasis | Absence | Reference | | 0.715 |  |  | NI |  | > 1 | 1.17 | 0.22-6.17 | 0.857 | 0.68 | 0.10-4.63 | 0.693 |
|  | Presence | 1.33 | 0.29-6.23 |  |  |  |  | PI | 0 | Reference | |  |  |  | NI |
| LN13 metastasis | Absence | Reference | | 0.277 |  |  | NI |  | 1 | 1.06 | 0.32-3.53 | 0.93 |  |  |  |
|  | Presence | 1.87 | 0.61-5.77 |  |  |  |  |  | 2 | 1.17 | 0.14-9.79 | 0.883 |  |  |  |
| LN14 metastasis | Absence | Reference | |  |  |  | NI | NLR | ≤ 3.32 | Reference | | 0.517 |  |  | NI |
|  | Presence | - |  |  |  |  |  |  | > 3.32 | 1.43 | 0.48-4.24 |  |  |  |  |
| LN15 metastasis | Absence | Reference | | 0.018 | Reference | | 0.030 | dNLR | ≤ 3.32 | Reference | | 0.832 |  |  | NI |
|  | Presence | 7.83 | 1.43-42.94 |  | 8.51 | 1.27-59.11 |  |  | > 3.32 | 0.89 | 0.29-2.72 |  |  |  |  |
| LN16 metastasis | Absence | Reference | | 0.196 |  |  | NI | PLR | ≤ 98.13 | Reference | | 0.577 |  |  | NI |
|  | Presence | 2.83 | 0.58-13.75 |  |  |  |  |  | > 98.13 | 1.80 | 0.23-14.17 |  |  |  |  |
| LN17 metastasis | Absence | Reference | |  |  |  | NI | PNI | 0 | Reference | | 0.993 |  |  | NI |
|  | Presence | - |  |  |  |  |  |  | 1 | 1.01 | 0.27-3.72 |  |  |  |  |
| LN18 metastasis | Absence | Reference | |  |  |  | NI | SII | ≤ 1000 | Reference | | 0.367 |  |  | NI |
|  | Presence | - |  |  |  |  |  |  | > 1000 | 1.65 | 0.56-4.90 |  |  |  |  |
| Positive LN number | 0 | Reference | |  |  |  | NI | mGPS | 0 | Reference | |  |  |  | NI |
|  | 1 - 3 | 2.94 | 0.84-10.34 | 0.092 |  |  |  |  | 1 | 1.73 | 0.56-5.35 | 0.342 |  |  |  |
|  | > 3 | 2.31 | 0.41-13.17 | 0.345 |  |  |  |  | 2 | - |  |  |  |  |  |
| Pancreatic membrane invasion | Absence | Reference | | 0.179 |  |  | NI | WBC | ≤ 10 | Reference | | 0.983 |  |  | NI |
|  | Presence | 0.41 | 0.11-1.50 |  |  |  |  |  | > 10 | 0.98 | 0.12-7.84 |  |  |  |  |
| Tumor size (cm) | ≤ 2 | Reference | |  |  |  | NI | ALB (g/L) | ≤ 35 | Reference | |  |  |  | NI |
|  | 2 – 4 | 1.43 | 0.36-5.57 | 0.613 |  |  |  |  | > 35 | - |  |  |  |  |  |
|  | > 4 | 1.77 | 0.38-8.19 | 0.465 |  |  |  | CRP (ng/L) | ≤ 3 | Reference | | 0.832 |  |  | NI |
| Tumor differentiation | Well | Reference | |  |  |  | NI |  | > 3 | 1.13 | 0.37-3.46 |  |  |  |  |
|  | Moderate | - |  |  |  |  |  | CA19-9 (U/ml) | ≤ 35 | Reference | | 0.387 |  |  | NI |
|  | Poor | - |  |  |  |  |  |  | > 35 | 0.59 | 0.18-1.95 |  |  |  |  |
| Macrovascular invasion | Absence | Reference | | 0.139 |  |  | NI | CEA (ng/ml) | ≤ 5 | Reference | | 0.386 |  |  | NI |
|  | Presence | 2.75 | 0.72-10.28 |  |  |  |  |  | > 5 | 0.56 | 0.15-2.07 |  |  |  |  |
| Microvascular invasion | Absence | Reference | | 0.367 |  |  | NI | HBV infection | Absence | Reference | | 0.893 |  |  | NI |
|  | Presence | 1.65 | 0.56-4.90 |  |  |  |  |  | Presence | 1.15 | 0.14-9.32 |  |  |  |  |
| Lymph vessel invasion | Absence | Reference | | 0.542 |  |  | NI | Chemotherapy | No | Reference | | 0.819 |  |  | NI |
|  | Presence | 1.24 | 0.57-2.54 |  |  |  |  |  | Yes | 0.88 | 0.30-2.58 |  |  |  |  |

Abbreviations as in Supplement Table 1

Supplement Table 4. Risk factors for multiple metastases in PDAC patients after surgery

| Characteristics | | Univariate analysis | | | Multivariate analysis | | | Characteristics | | Univariate analysis | | | Multivariate analysis | | |
| --- | --- | --- | --- | --- | --- | --- | --- | --- | --- | --- | --- | --- | --- | --- | --- |
|  |  | HR | 95% | P | HR | 95% | P |  |  | HR | 95% | P | HR | 95% | P |
| Age | ≤ 60 years | Reference | | 0.031 | Reference | | 0.033 | Perineural invasion | Absence | Reference | | 0.812 |  |  | NI |
|  | > 60 years | 10.03 | 1.24-81.23 |  | 9.82 | 1.20-80.66 |  |  | Presence | 1.18 | 0.31-4.47 |  |  |  |  |
| Gender | Female | Reference | | 0.298 |  |  | NI | Adjacent organ invasion | Absence | Reference | | 0.266 |  |  | NI |
|  | Male | 2.33 | 0.48-11.40 |  |  |  |  |  | Presence | 2.51 | 0.50-12.61 |  |  |  |  |
| LN metastasis | Absence | Reference | | 0.579 |  |  | NI | LNR | 0 | Reference | |  |  |  | NI |
|  | Presence | 0.67 | 0.17-2.74 |  |  |  |  |  | 0 - 0.16 | 1.16 | 0.23-5.89 | 0.859 |  |  |  |
| LN5 metastasis | Absence | Reference | |  |  |  | NI |  | > 0.16 | 0.91 | 0.38-2.21 | 0.838 |  |  |  |
|  | Presence | - |  |  |  |  |  | Satellite foci | Absence | Reference | |  |  |  | NI |
| LN6 metastasis | Absence | Reference | |  |  |  | NI |  | Presence | - |  |  |  |  |  |
|  | Presence | - |  |  |  |  |  | Tumor site | Head | Reference | | 0.731 |  |  | NI |
| LN7 metastasis | Absence | Reference | | 0.087 |  |  | NI |  | Body and tail | 1.32 | 0.27-6.56 |  |  |  |  |
|  | Presence | 7.20 | 0.75-68.93 |  |  |  |  | Imaging tumor size (cm) | ≤ 2 | Reference | |  |  |  | NI |
| LN8 metastasis | Absence | Reference | | 0.148 |  |  | NI |  | 2 – 4 | 0.73 | 0.15-3.70 | 0.706 |  |  |  |
|  | Presence | 5.11 | 0.56-46.55 |  |  |  |  |  | > 4 | 1.87 | 0.37-9.59 | 0.453 |  |  |  |
| LN9 metastasis | Absence | Reference | | 0.218 |  |  | NI | Imaging LN metastasis | Absence | Reference | | 0.593 |  |  | NI |
|  | Presence | 3.94 | 0.45-34.97 |  |  |  |  |  | Presence | 0.68 | 0.17-2.78 |  |  |  |  |
| LN10 metastasis | Absence | Reference | |  |  |  | NI | Imaging vascular invasion | Absence | Reference | | 0.970 |  |  | NI |
|  | Presence | - |  |  |  |  |  |  | Presence | 0.97 | 0.20-4.78 |  |  |  |  |
| LN11 metastasis | Absence | Reference | |  |  |  | NI | Imaging LN size (cm) | ≤ 0.5 | Reference | |  |  |  | NI |
|  | Presence | - |  |  |  |  |  |  | 0.5-1 | 0.39 | 0.05-3.20 | 0.377 |  |  |  |
| LN12 metastasis | Absence | Reference | | 0.989 |  |  | NI |  | > 1 | 0.41 | 0.05-3.36 | 0.402 |  |  |  |
|  | Presence | 0.99 | 0.12-8.13 |  |  |  |  | PI | 0 | Reference | |  |  |  | NI |
| LN13 metastasis | Absence | Reference | | 0.926 |  |  | NI |  | 1 | 1.19 | 0.29-4.88 | 0.81 |  |  |  |
|  | Presence | 0.93 | 0.19-4.57 |  |  |  |  |  | 2 | - |  |  |  |  |  |
| LN14 metastasis | Absence | Reference | | 0.007 | Reference | | 0.010 | NLR | ≤ 3.32 | Reference | | 0.927 |  |  | NI |
|  | Presence | 7.64 | 1.76-33.08 |  | 7.38 | 1.61-33.74 |  |  | > 3.32 | 0.94 | 0.23-3.82 |  |  |  |  |
| LN15 metastasis | Absence | Reference | |  |  |  | NI | dNLR | ≤ 3.32 | Reference | | 0.99 |  |  | NI |
|  | Presence | - |  |  |  |  |  |  | > 3.32 | 0.99 | 0.24-4.04 |  |  |  |  |
| LN16 metastasis | Absence | Reference | | 0.516 |  |  | NI | PLR | ≤ 98.13 | Reference | | 0.94 |  |  | NI |
|  | Presence | 2.03 | 0.24-17.18 |  |  |  |  |  | > 98.13 | 1.09 | 0.13-8.94 |  |  |  |  |
| LN17 metastasis | Absence | Reference | |  |  |  | NI | PNI | 0 | Reference | | 0.388 |  |  | NI |
|  | Presence | - |  |  |  |  |  |  | 1 | 0.54 | 0.13-2.21 |  |  |  |  |
| LN18 metastasis | Absence | Reference | |  |  |  | NI | SII | ≤ 1000 | Reference | | 0.413 |  |  | NI |
|  | Presence | - |  |  |  |  |  |  | > 1000 | 1.75 | 0.46-6.66 |  |  |  |  |
| Positive LN number | 0 | Reference | |  |  |  | NI | mGPS | 0 | Reference | |  |  |  | NI |
|  | 1 - 3 | 0.63 | 0.12-3.32 | 0.288 |  |  |  |  | 1 | 0.98 | 0.11-8.41 | 0.985 |  |  |  |
|  | > 3 | 0.89 | 0.10-7.88 | 0.917 |  |  |  |  | 2 | 0.99 | 0.09-11.27 | 0.990 |  |  |  |
| Pancreatic membrane invasion | Absence | Reference | | 0.721 |  |  | NI | WBC | ≤ 10 | Reference | |  |  |  | NI |
|  | Presence | 0.77 | 0.19-3.16 |  |  |  |  |  | > 10 | - |  |  |  |  |  |
| Tumor size (cm) | ≤ 2 | Reference | |  |  |  | NI | ALB (g/L) | ≤ 35 | Reference | | 0.557 |  |  | NI |
|  | 2 – 4 | 0.80 | 0.17-3.65 | 0.771 |  |  |  |  | > 35 | 0.62 | 0.12-3.08 |  |  |  |  |
|  | > 4 | 0.86 | 0.14-5.29 | 0.869 |  |  |  | CRP (ng/L) | ≤ 3 | Reference | | 0.989 |  |  | NI |
| Tumor differentiation | Well | Reference | |  |  |  | NI |  | > 3 | 1.01 | 0.25-4.13 |  |  |  |  |
|  | Moderate | - |  |  |  |  |  | CA19-9 (U/ml) | ≤ 35 | Reference | | 0.525 |  |  | NI |
|  | Poor | - |  |  |  |  |  |  | > 35 | 1.97 | 0.24-16.10 |  |  |  |  |
| Macrovascular invasion | Absence | Reference | | 0.211 |  |  | NI | CEA (ng/ml) | ≤ 5 | Reference | | 0.937 |  |  | NI |
|  | Presence | 2.82 | 0.56-14.23 |  |  |  |  |  | > 5 | 1.06 | 0.26-4.33 |  |  |  |  |
| Microvascular invasion | Absence | Reference | | 0.920 |  |  | NI | HBV infection | Absence | Reference | | 0.552 |  |  | NI |
|  | Presence | 1.08 | 0.26-4.39 |  |  |  |  |  | Presence | 1.91 | 0.23-16.11 |  |  |  |  |
| Lymph vessel invasion | Absence | Reference | | 0.577 |  |  | NI | Chemotherapy | No | Reference | | 0.875 |  |  | NI |
|  | Presence | 1.36 | 0.57-5.46 |  |  |  |  |  | Yes | 0.90 | 0.24-3.41 |  |  |  |  |

Abbreviations as in Supplement Table 1
